# Supplementary material for: Sphingomyelin-derived nanovesicles for the delivery of the IDO1 inhibitor epacadostat enhance metastatic and post-surgical melanoma immunotherapy
Source: Nat Commun. 2023 Nov 9;14:7235. doi: 10.1038/s41467-023-43079-4 (PMC10636136; doi:10.1038/s41467-023-43079-4)
Supplement: Supplementary file 1 — Supplementary Information [file 41467_2023_43079_MOESM1_ESM.pdf]

Supplementary information

Sphingomyelin-derived nanovesicles for the delivery of the IDO1 inhibitor epacadostat enhance metastatic and post-surgical melanoma immunotherapy

Zhiren Wang[1], Wenpan Li[1], Yanhao Jiang[1], Tuyen Ba Tran[1], Leyla Estrella Cordova[1], Jinha Chung[1], Minhyeok Kim[1], Georg Wondrak[1,2], Jennifer Erdrich[3], Jianqin Lu[1,2,4,5]*

[1]Skaggs Pharmaceutical Sciences Center, Department of Pharmacology & Toxicology, R. Ken Coit College of Pharmacy, The University of Arizona, Tucson, Arizona, 85721, United States

[2]NCI-designated University of Arizona Comprehensive Cancer Center, Tucson, Arizona, 85721, United States

[3]Department of Surgery, Division of Surgical Oncology, The University of Arizona College of Medicine, Tucson, Arizona, 85721, United States

[4]BIO5 Institute, The University of Arizona, Tucson, Arizona, 85721, United States

[5]Southwest Environmental Health Sciences Center, The University of Arizona, Tucson, 85721, United States

Address correspondence to: Jianqin Lu, B.Pharm., Ph.D.

Assistant Professor & Director

Pharmaceutics & Pharmacokinetics Track

Skaggs Pharmaceutical Sciences Center 422

Department of Pharmacology & Toxicology

R. Ken Coit College of Pharmacy

The University of Arizona

1703 East Mabel Street, Tucson, AZ 85721

Tel: 520-626-1786; Fax: 520-626-2466

Email: lu6@arizona.edu

Supplementary Methods

Materials

Di(1*H*-imidazol-1-yl)methanone (CDI, 98%), Dacarbazine (DTIC, 98%) and Epacadostat (EPA, 98%) were purchased from BLDpharm (Shanghai, China). Sphingomyelin (SM, egg, 99%), 1,2-distearoyl-sn-glycero-3-phosphoethanolamine-N-[methoxy(polyethylene glycol)-2000] ammonium salt (DSPE-PEG_{2K}, 99%) and cholesterol (Chol, 99%) were purchased from Avanti (Alabama, USA). Succinic anhydride (98%), N, N-diisopropylethylamine (98%), esterase from horse liver and 4-pyrrolidinopyridine (4-PPY, 98%) were purchased from Sigma-Aldrich (MO, USA). Trypsin-EDTA solution, Triton X-100, and Dulbecco's Modified Eagle's Medium (DMEM), fetal bovine serum (FBS) and penicillin-streptomycin solution were all purchased from Gibco (MD, USA). All solvents used for chemical reactions were anhydrous, and the eluting solvents for compound purification were HPLC grade.

Chemical synthesis

The NMR spectra were acquired by Bruker topspin software (v. 2.1) using TMS (0 ppm) as the internal standard on a AVIII 500 MHz spectrometer and analysed by MestReNova (v. 6.0.2). ^{1}H NMR data were reported as follows: chemical shift, multiplicity (s = singlet, d = doublet, m = multiplet), coupling constant in Hertz (Hz) and hydrogen numbers based on integration intensities. ^{13}C NMR chemical shifts are reported in ppm relative to the central peak of TMS (0 ppm) as internal standards. The high-resolution mass spectra (HRMS) were generated via an LTQ Orbitrap Velos mass spectrometer with an ESI source (Thermo Scientific). The low-resolution mass spectra were generated using a LCMS-2020 + DUIS-2020 (Shimadzu). The reactions were followed by thin-layer chromatography (TLC, Silica gel 60 F_{254}, Merck KGaA) on glass-packed precoated silica gel plates and visualized in an iodine chamber or with a UV lamp. Flash column chromatography was performed using silica gel (SiliaFlash® P60, 230–400 mesh) purchased from Silicycle Inc.

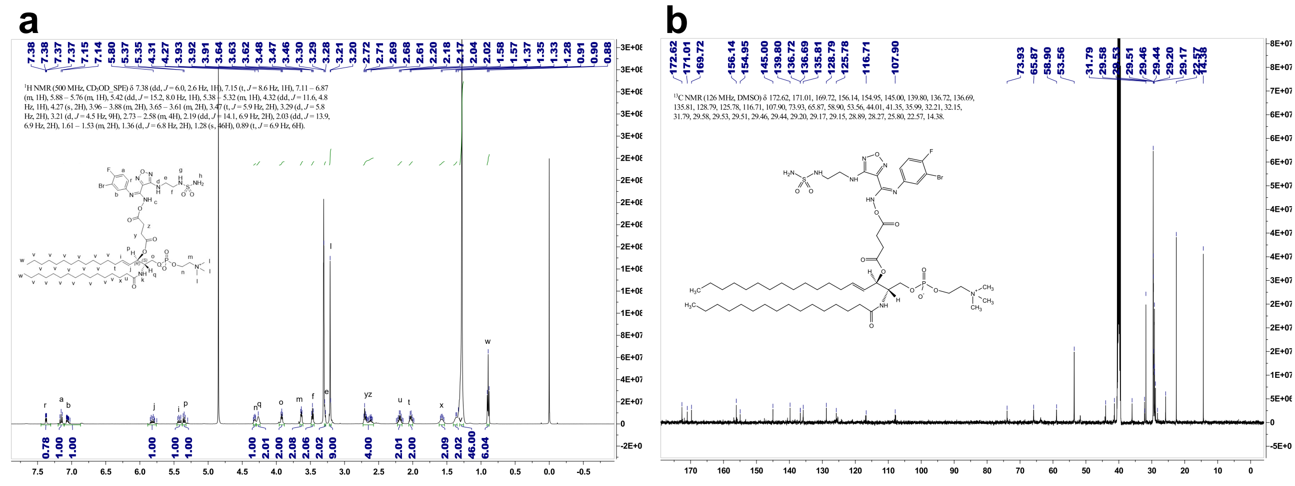

Supplementary Figure 1. ^{1}H NMR (**a**) and ^{13}C NMR (**b**) spectra for SM-EPA.

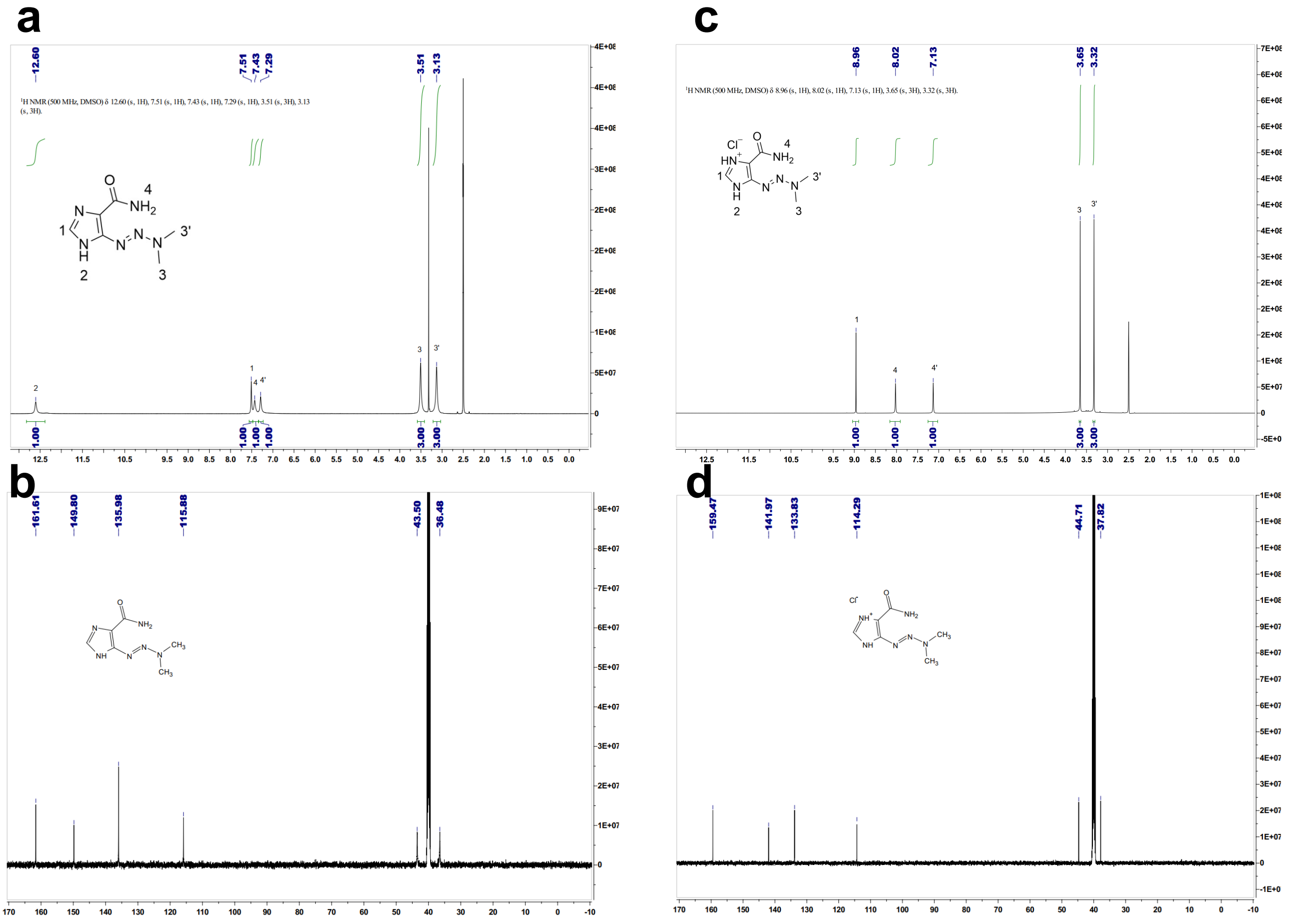

Supplementary Figure 2. ^{1}H NMR and ^{13}C NMR spectra for free DTIC (**a, b**) and DTIC hydrochloride (**c, d**), respectively.

a HPLC for EPA

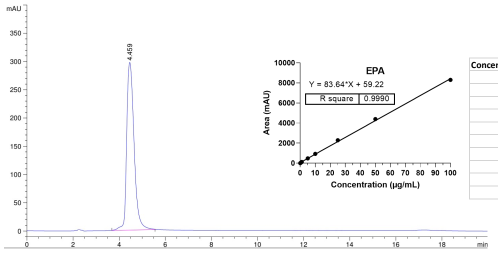

Concentration (μg/mL)	Area (mAU)
0.05	4.12
0.1	8.41
0.25	45.45
0.5	58.85
1	122.368
5	482.746
10	929.741
25	2283.58
50	4399.72
100	8307.19

Instrumentation	
**HPLC Unit**	Agilent 1100 (USA)
**Detector**	Agilent G1315B Diode-Array Detector
**Integrator**	Agilent ChemStation Software
**Chromatographic conditions**	
**Column**	Waters Symmetry ODS (250mm X 4 mm X 5 μm)
**Mobile Phase**	Acetonitrile : NH_4Ac (15 mM) (30:70 v/v)
**Flow Rate**	1 mL/min
**Injection Volume**	50 μL
**$λ_{max}$**	254 nm

b HPLC for SM-EPA

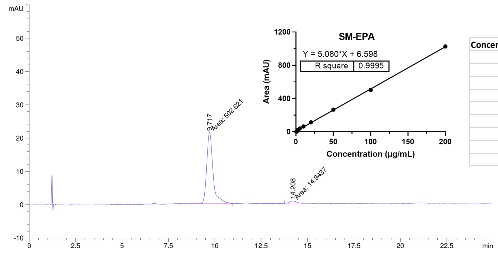

Concentration (μg/mL)	Area (mAU)
0.5	1
1	2.3
2.5	21.3
5	39.87
10	63.5
20	113.2
50	265.3
100	502.55
200	1026.5

Instrumentation	
**HPLC Unit**	Agilent 1100 (USA)
**Detector**	Agilent G1315B Diode-Array Detector
**Integrator**	Agilent ChemStation Software
**Chromatographic conditions**	
**Column**	Waters Symmetry ODS (250mm X 4 mm X 5 μm)
**Mobile Phase**	Acetonitrile : Methanol : H_2O (15 mM) (15:80:5 v/v)
**Flow Rate**	2 mL/min
**Injection Volume**	50 μL
**$λ_{max}$**	254 nm

Supplementary Figure 3. The Analytic Reverse-phase High Performance Liquid Chromatography (HPLC) method development for free EPA (**a**) and SM-EPA (**b**) concentration measurement in pharmacokinetics and biodistribution studies. Representative HPLC chromatogram, standard curve, and HPLC instrumentation and chromatographic conditions. Source data are provided as a Source Data file.

HPLC for DTIC

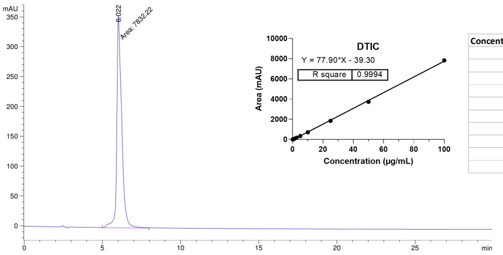

Concentration (µg/mL)	Area (mAU)
0.1	5.175
0.25	15.23
0.5	31.38
1	65.42
2.5	170.21
5	338.87
10	712.83
25	1847.94
50	3727.96
100	7832.22

Instrumentation	
**HPLC Unit**	Agilent 1100 (USA)
**Detector**	Agilent G1315B Diode-Array Detector
**Integrator**	Agilent ChemStation Software
**Chromatographic conditions**	
**Column**	Waters Symmetry ODS (250mm X 4 mm X 5 µm)
**Mobile Phase**	Acetonitrile : NH_4Ac (15 mM) (30:70 v/v)
**Flow Rate**	1 mL/min
**Injection Volume**	50 µL
λ_{max}	254 nm

Supplementary Figure 4. The Analytic Reverse-phase High Performance Liquid Chromatography (HPLC) method development for DTIC concentration measurement in pharmacokinetics and biodistribution studies. Representative HPLC chromatogram, standard curve, and HPLC instrumentation and chromatographic conditions. Source data are provided as a Source Data file.

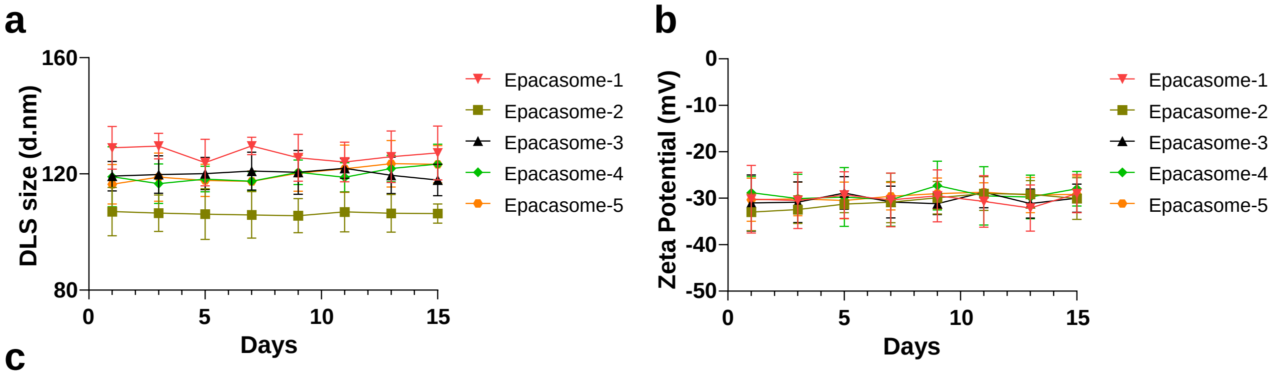

c

Liposomes	Lipid ratio	EPA loading efficiency (%)	EPA loading capacity (weight %)	DLS Size by intensity (d.nm)	Zeta Potential (mV)	Polydispersity Index (PDI)
**Lipo-SM/Chol (empty liposome)**	SM:Chol:DSPE-PEG$_{2K}$ = 82.82/12.78/4.40 (molar ratio)	NA	NA	120.2 ± 3.0	-10.3 ± 2.6	0.110 ± 0.024
**EPA/Lipo-SM/Chol**	SM:Chol:DSPE-PEG$_{2K}$ = 82.82/12.78/4.40 (molar ratio)	72.3	1.27	154.5 ± 7.5	-18.5 ± 4.8	0.114 ± 0.016
**EPA/Lipo-DOPE/Chol**	DOPE:DOTAP:Chol:DSPE-PEG$_{2K}$ = 9/9/2/1 (weight ratio)	62.1	1.08	126.6 ± 4.9	-16.6 ± 6.8	0.228 ± 0.018
**EPA/Lipo-HSPC/Chol**	HSPC:Chol:DSPE-PEG$_{2K}$ = 22.3/7.3/7.3 (weight ratio)	53.6	0.23	117.7 ± 5.0	-12.6 ± 3.6	0.115 ± 0.010

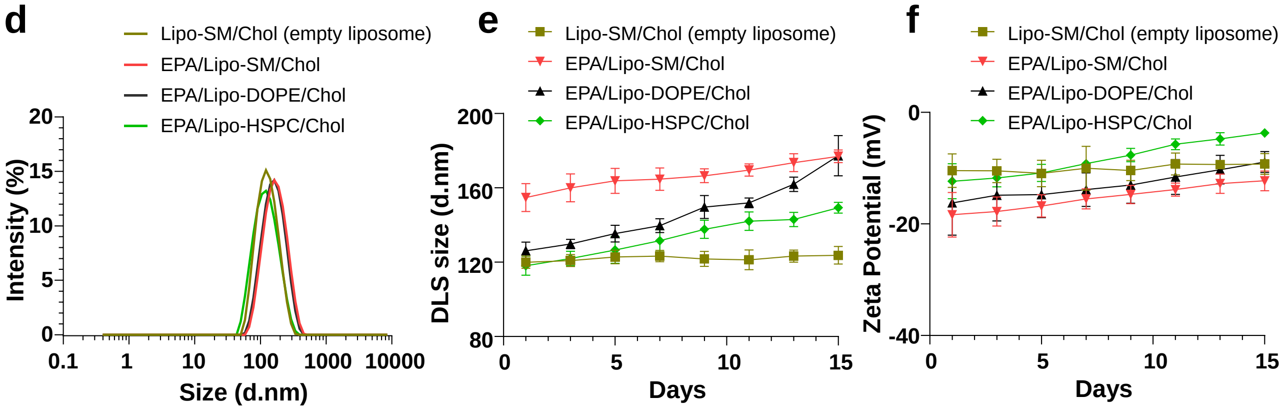

Supplementary Figure 5. The DLS Size (**a**) and zeta potential (**b**) of Epacasome monitoring over a 15-day period at 4 °C. **c**, A table illustrating the physicochemical properties of Lipo-SM/Chol (empty liposome), EPA/Lipo-SM/Chol, EPA/Lipo-DOPE/Chol and EPA/Lipo-HSPC/Chol with detailed ratios[1,2]. d.nm, diameter values in nanometres. DLS, dynamic light scattering. **d**, DLS size distribution by intensity. The DLS Size (**e**) and zeta potential (**f**) of various liposomes monitoring over a 15-day period at 4 °C. Data in **a**, **b**, **c**, **e** and **f** are represented as mean ± s.d. (n = 3 independent experiments). Source data are provided as a Source Data file.

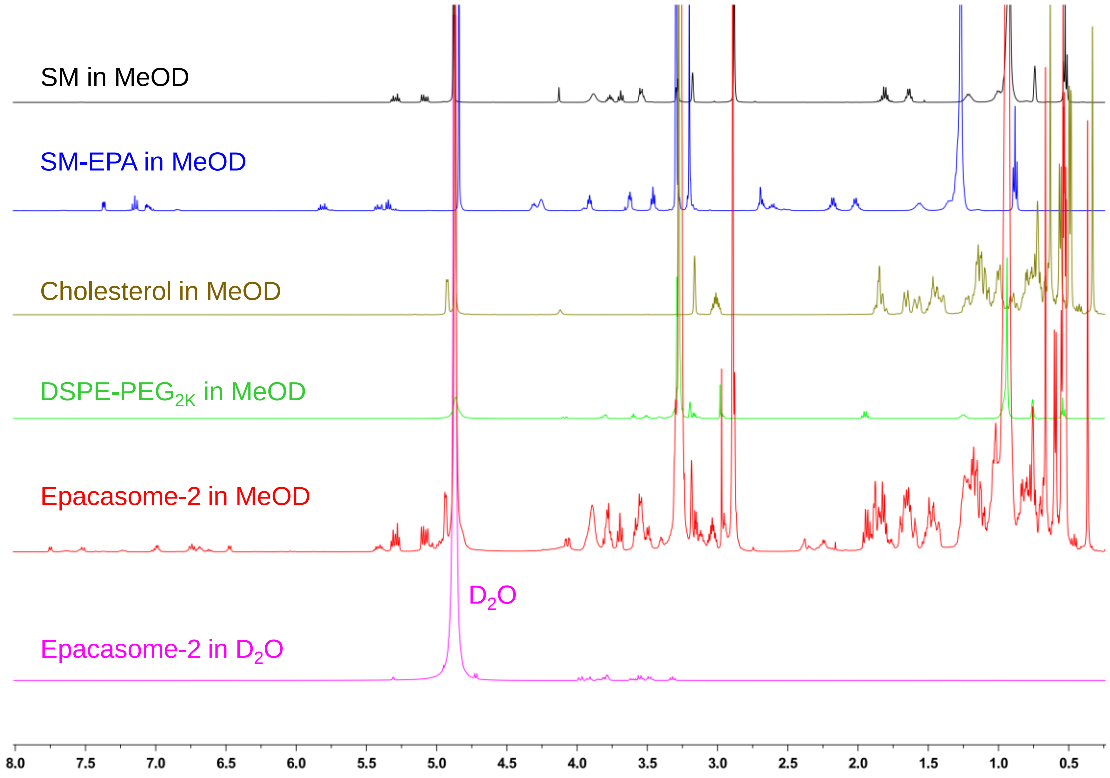

Supplementary Figure 6. ^{1}H NMR spectra of Epacasome-2 in D_2O, and SM-EPA conjugate, SM, Cholesterol, and DSPE-PEG_{2K} in MeOD. The typical proton spectra were shown for SM-EPA, SM, Cholesterol, DSPE-PEG_{2K} in MeOD due to their free dispersion in this solvent. Epacasome-2 in MeOD expressed all typical proton signals for each individual constituent. However, when collected in D_2O, nearly all the proton signals from individual components in Epacasome-2 were all suppressed, which can be attributed to their spontaneous self-assembly into Epacasome-2.

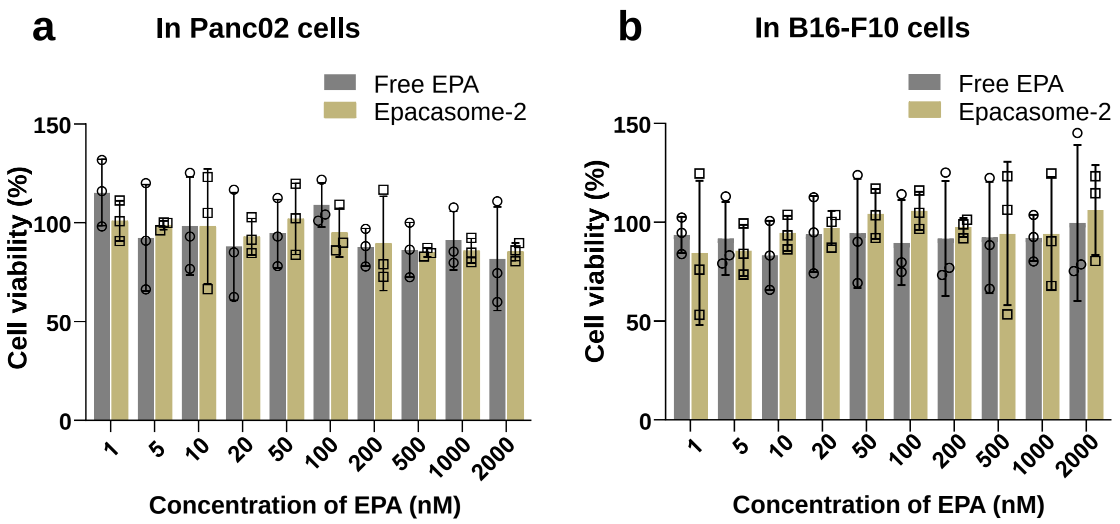

Supplementary Figure 7. The cytotoxicity of free EPA and Epacasome-2 in Panc02 (**a**) and B16-F10 (**b**) cells. Cells were treated for 72 h and cell viability was determined by MTT assay. Data in **a**, **b** are represented as mean ± s.d. (n = 3 biologically independent samples). Source data are provided as a Source Data file.

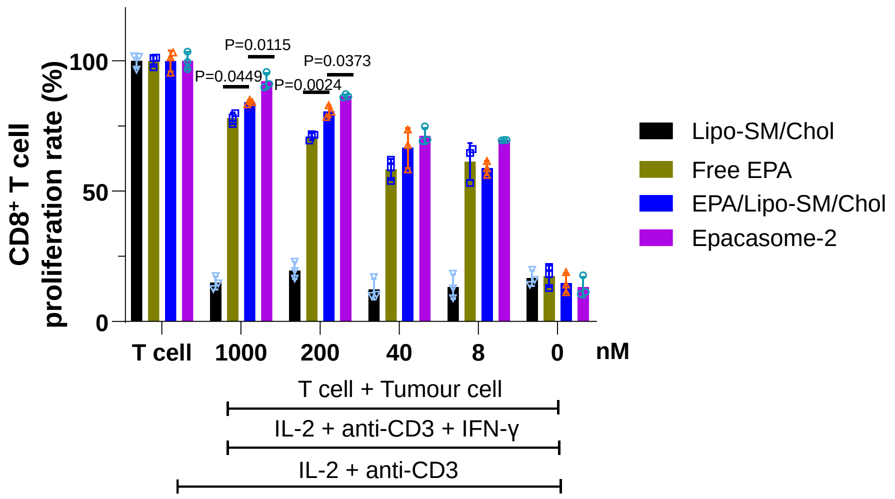

Supplementary Figure 8. CD8$^+$ T cell proliferation by co-culture for 96 h. B16-F10 cells were stimulated by IFN-γ to induce IDO1 expression, then treated by Mitomycin C prior to mixing with splenocytes. Free EPA, Lipo-SM/Chol, EPA/Lipo-SM/Chol and Epacasome-2 were added to co-culture cells at eq. dose of EPA (0, 8, 40, 200, and 1000 nM). To evaluate T cell proliferation, anti-CD3 and IL-2 were added to co-cultures. 4 days later, CD8$^+$ T cell proliferation was assessed by FACS analysis. Data are expressed as mean ± s.d (n = 3 independent experiments). Statistical significance was determined by one-way ANOVA followed by Tukey's multiple comparisons test. Source data are provided as a Source Data file.

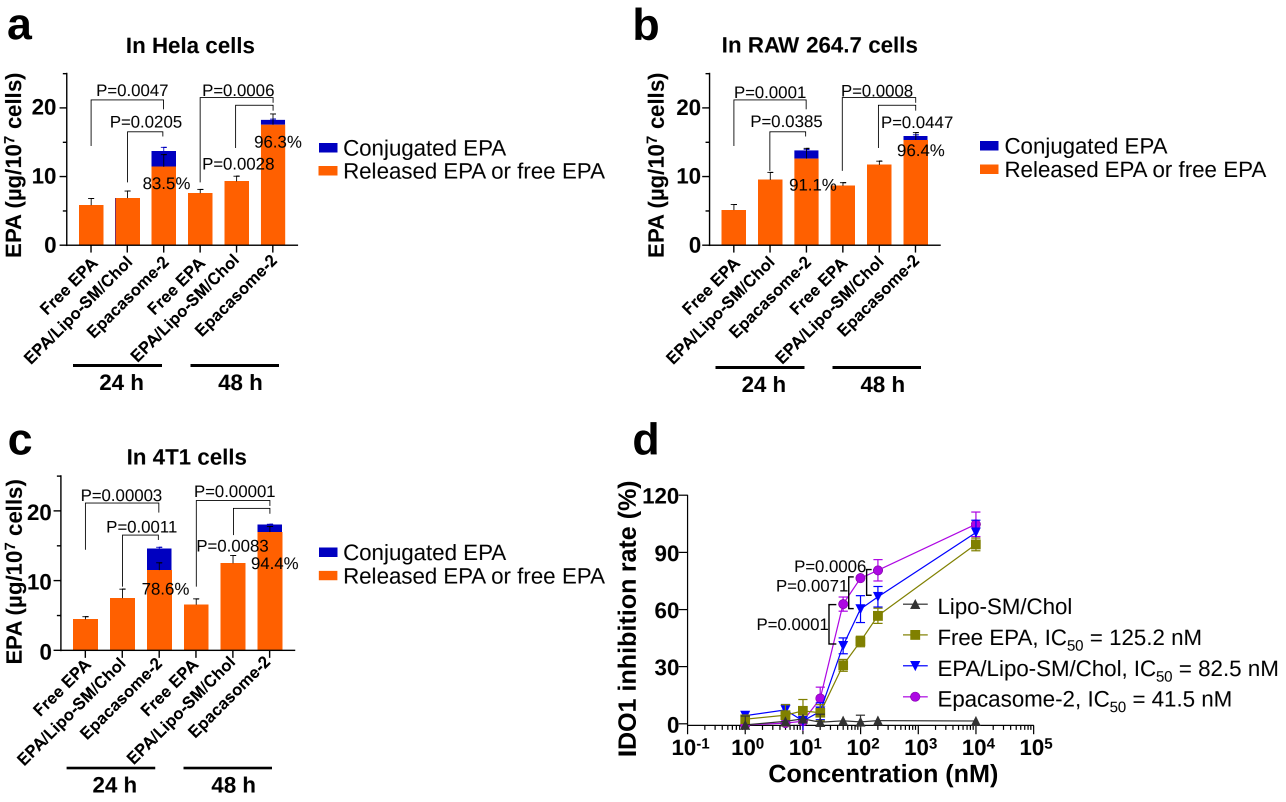

Supplementary Figure 9. Cellular uptake levels and EPA release of Epacasome-2 in Hela (**a**), macrophages RAW 264.7 (**b**) and 4T1 (**c**) cells after 24 and 48 h incubation measured by HPLC, respectively. The release ratios of EPA from Epacasome-2 were presented as percentage values in the figures. **d**, IDO1 enzymatic inhibitory activity via measuring the Kyn in supernatants in 4T1 cells treated with IFN-γ along with free EPA, Lipo-SM/Chol, EPA/Lipo-SM/Chol and Epacasome-2 at equivalent (eq.) EPA concentration for 48 h. Data in **a-d** are expressed as mean ± s.d (n = 3 independent experiments). Statistical significance was determined by one-way ANOVA followed by Tukey's multiple comparisons test. Source data are provided as a Source Data file.

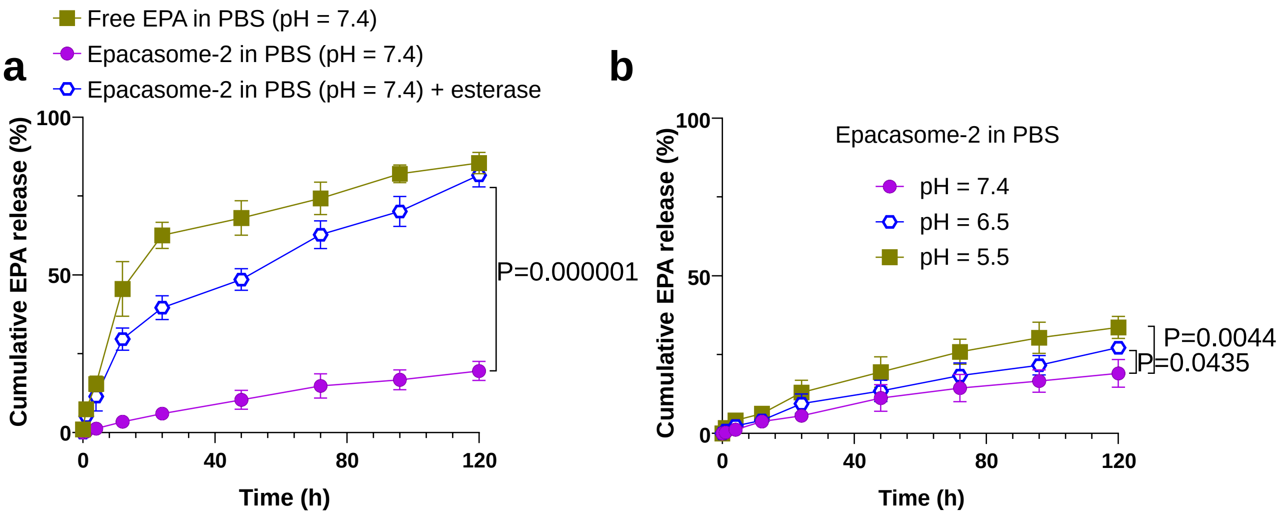

Supplementary Figure 10. The EPA release kinetics of Epacasome-2 under PBS and 10 units of esterase (**a**) and different pH conditions (**b**) at 37 °C. Data are represented as mean ± s.d. (n = 3 independent experiments). Statistical significance was determined by one-way ANOVA followed by Tukey's multiple comparisons test. Source data are provided as a Source Data file.

a

Formulation	DSPE-Cy5 (w/w%)	DLS size by intensity (d.nm)	Zeta potential (mV)	PDI
**Cy5/Epacasome-2**	0.2%	105.1 ± 7.1	-31.5 ± 5.2	0.108 ± 0.054
**Cy5/EPA/Lipo-SM/Chol**	0.2%	151.3 ± 4.6	-19.3 ± 4.3	0.162 ± 0.071

b

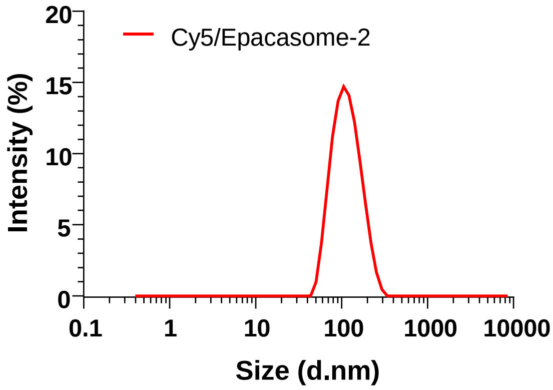

c

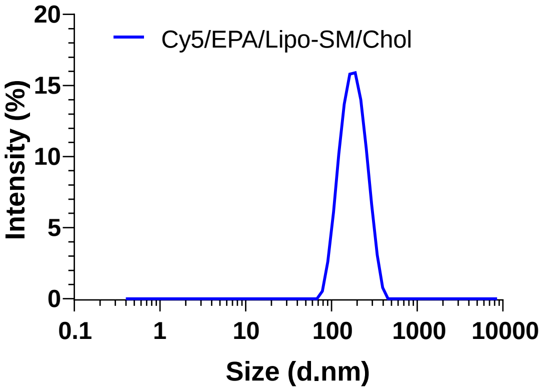

Supplementary Figure 11. Development and physicochemical characterizations of Cy5-labeled- Epacasome-2. **a,** A table shows the physicochemical characterizations of Cy5/Epacasome-2 and Cy5/EPA/Lipo-SM/Chol with regards to size, zeta potential and polydispersity using 0.2 weight % of DSPE-Cy5. The representative DLS size distribution by intensity for Cy5/Epacasome-2 (**b**) and Cy5/EPA/Lipo-SM/Chol (**c**). Data in **a** are represented as mean ± s.d. (n = 3 independent experiments). Source data are provided as a Source Data file.

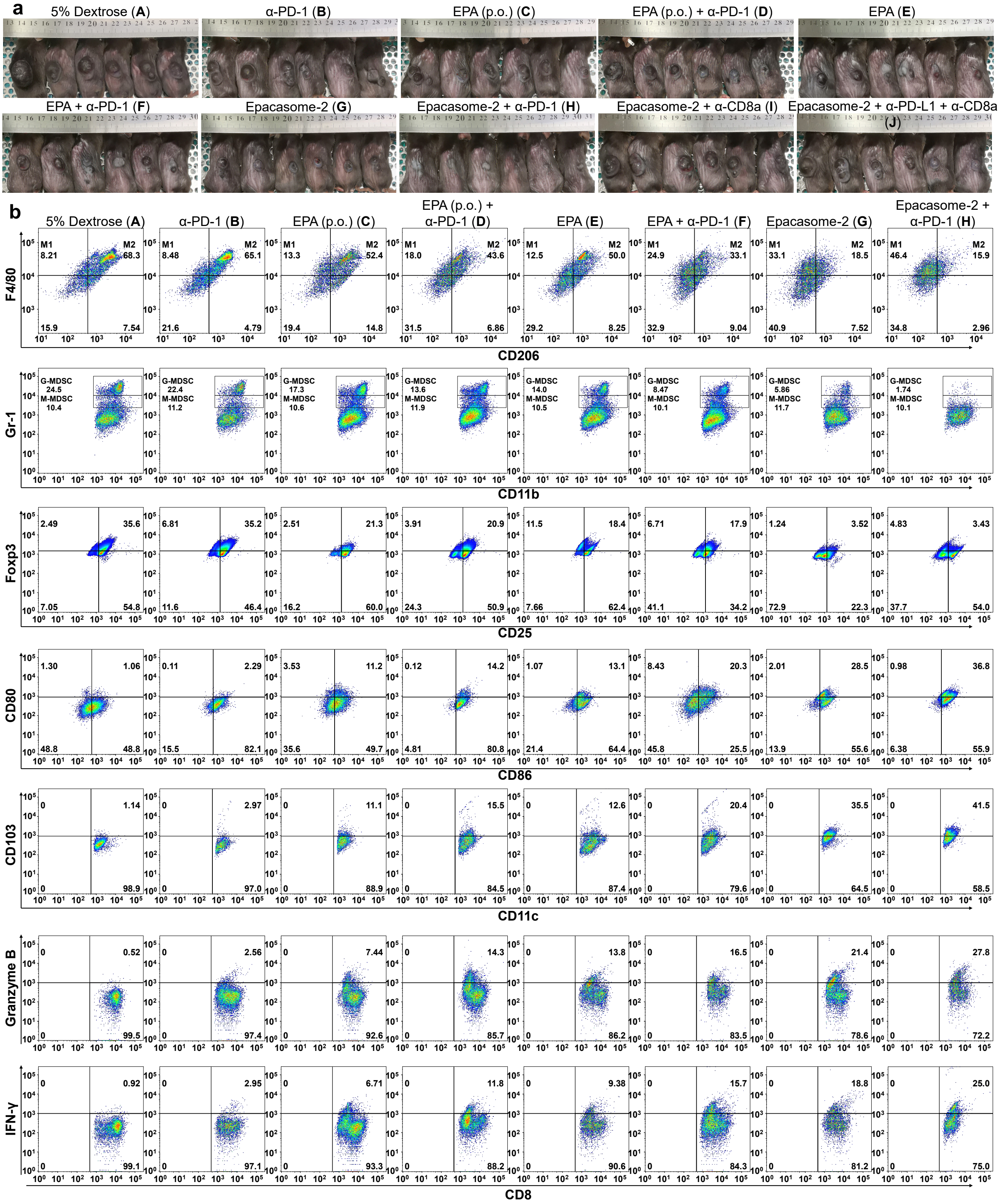

Supplementary Figure 12. a, Mice bearing s.c. B16-F10 tumour images taken on day 15 from **Fig. 3** (n = 6 mice). **b**, Representative flow cytometric analysis of intratumoural CD45^{+}/CD11b^{+}/F4/80^{+}/CD206^{-} M1, and CD45^{+}/CD11b^{+}/F4/80^{+}/CD206^{+} M2 TAMs cells[3,4], CD45^{+}/CD11b^{+}/Gr-1^{+} MDSC cells, CD3^{+}/CD4^{+}/Foxp3^{+}/CD25^{+} Tregs, CD45^{+}/CD11c^{+}/CD80^{+}/CD86^{+} DCs, CD45^{+}/CD11c^{+}/CD103^{+} DCs, Granzyme B^{+} or IFN-γ^{+}/CD8^{+} T cells for **Fig. 4f-n**.

Group (n = 6 mice)	Median survival time (**MST**, days)	Time to reach endpoint (**TTE**, days)						Tumour growth delay (**TGD**, days)						Increased live span (**ILS**, %)					
5% Dextrose	17.5	16	18	17	15	22	21	NA	NA	NA	NA	NA	NA	NA	NA	NA	NA	NA	NA
α-PD-1	19	17	20	19	20	19	17	1.2	2.8	2.3	2.8	1.8	1.1	-2.9%	14.3%	8.6%	14.3%	8.6%	-2.9%
EPA (p.o.)	20	19	20	22	20	18	21	2.9	3.4	3.8	2.7	2.7	5.5	8.6%	14.3%	25.7%	14.3%	2.9%	20.0%
EPA (p.o.) + α-PD-1	22.5	19	21	24	23	25	22	2.8	5.2	8.4	1.6	9	6.2	8.6%	20.0%	37.1%	31.4%	42.9%	25.7%
EPA	21.5	25	21	21	22	23	21	6.9	4.8	4.4	5.7	6.9	5	42.9%	20.0%	20.0%	25.7%	31.4%	20.0%
EPA + α-PD-1	24	23	25	26	28	22	22	5.8	9	9	11.2	5.3	6.2	31.4%	42.9%	48.6%	60.0%	25.7%	25.7%
Epacasome-2	27	26	27	29	27	26	28	10.9	12	13.5	11.3	11.4	13	48.6%	54.3%	65.7%	54.3%	48.6%	60.0%
Epacasome-2 + α-PD-1	31	30	29	31	32	31	34	15	13.7	15	16.7	16	18.4	71.4%	65.7%	77.1%	82.9%	77.1%	94.3%
Epacasome-2 + α-CD8a	18.5	22	18	21	17	16	19	4.9	0.3	0.8	0.7	0.9	0	25.7%	2.9%	20.0%	-2.9%	-8.6%	8.6%
Epacasome-2 + α-PD-L1 + α-CD8a	19	23	17	19	19	20	18	2.9	2.3	3.6	0.4	0.3	3.3	31.4%	-2.9%	8.6%	8.6%	14.3%	2.9%
**Statistical significance comparison**		P value by one-way ANOVA followed by Tukey's multiple comparisons test																	
EPA (p.o.) vs Epacasome-2		0.000003						0.0000000001						0.0000002					
EPA vs Epacasome-2		0.0021						0.0000004						0.0004					
EPA (p.o.) + α-PD-1 vs Epacasome-2 + α-PD-1		0.00000001						0.000000000000001						0.0000000006					
EPA + α-PD-1 vs Epacasome-2 + α-PD-1		0.000008						0.0000000001						0.0000007					
Epacasome-2 vs Epacasome-2 + α-PD-1		0.0286						0.0063						0.0089					

Supplementary Table 1. A table shows the median survival time (MST), time to reach endpoint (TTE), tumour growth delay (TGD) and increased live span (ILS) from **Fig. 4** (n = 6 mice). Statistical significance was determined by one-way ANOVA followed by Tukey's multiple comparisons test. Source data are provided as a Source Data file.

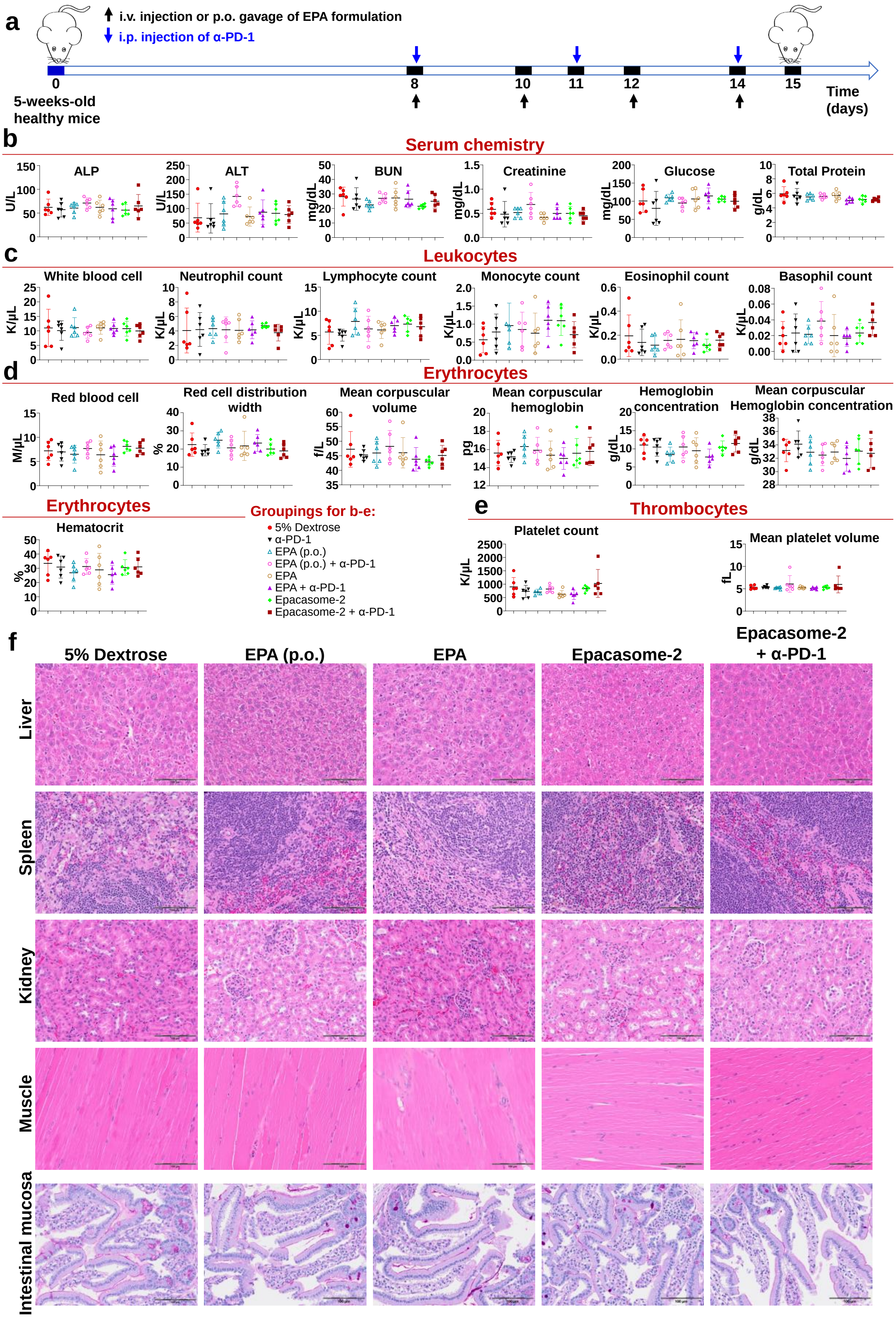

Supplementary Figure 13 a, Drug administration timeline scheme in healthy C57BL/6 mice that received the same treatments as **Fig. 4a**. **b-f**, On day 15, blood was withdrawn for

comprehensive serum chemistry (**b**), leukocytes (**c**), erythrocytes (**d**) and thrombocytes (**e**) analysis, and the liver, spleen, kidneys and muscle were isolated from the mice for hematoxylin & eosin (H&E) staining, and intestinal mucosa was stained by periodic acid-Schiff (PAS) reaction and counterstained with haematoxylin. Representative H&E staining of liver, spleen, kidneys and muscle and PAS staining of intestine mucosa, scale bar = 100 μm (**f**, n = 6 mice, similar results were observed). Data in **b-e** are expressed as mean ± s.d. (n = 6 mice). Statistical significance was determined by one-way ANOVA followed by Tukey's multiple comparisons test. Source data are provided as a Source Data file.

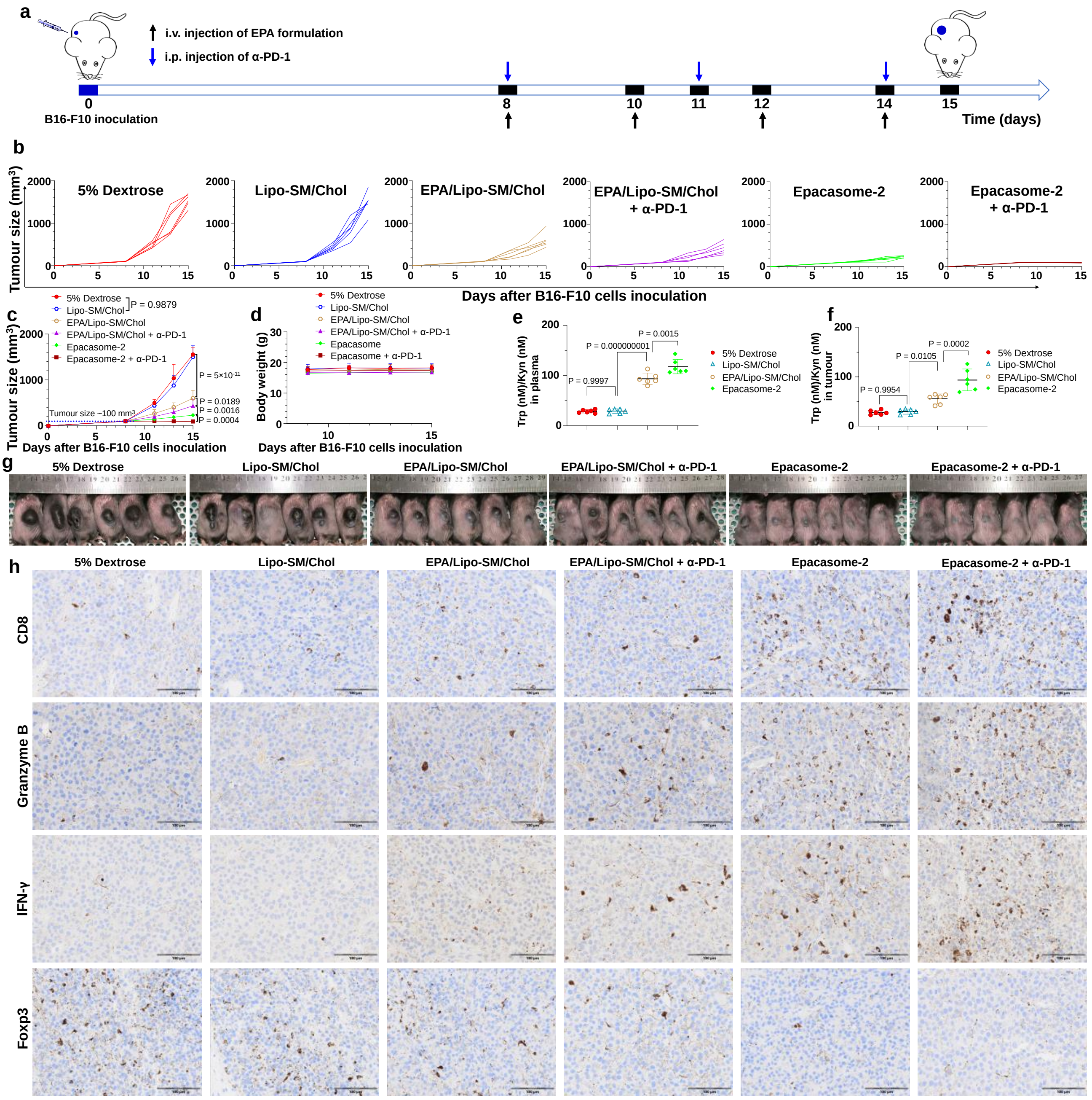

Supplementary Figure 14. a, Drug administration timeline scheme of antitumour efficacy in subcutaneous (s.c.) B16-F10 tumour model (n = 6 mice, tumours: ~100 mm^3), mice i.v. injected with Lipo-SM/Chol (empty liposome), EPA/Lipo-SM/Chol, Epacasome-2 at eq. 41 mg EPA/kg on day 8, 10, 12 and 14 alone or combined with i.p. α-PD-1 (BioXCell, clone RMP1-14, 100 µg per mouse per 3 day for 3 times) from day 8. **b**, Individual tumour growth curves. **c-d**, Average tumour size growth curves (**c**) and body weight (**d**). **e**,**f**, The ratio of Trp (nM)/kyn (nM) concentration in plasma (**e**) and tumours (**f**). **g**, Mice bearing s.c. B16-F10 tumour images taken on day 15. (**h**) Representative IHC staining of intratumoural CD8, Granzyme B, IFN-γ, and Foxp3 on day 15, scale bar = 100 µm (n = 6 mice, similar results were observed). Data in **c-f** are expressed as mean ± s.d (n = 6 mice). Statistical significance was determined by one-way ANOVA followed by Tukey's multiple comparisons test. Source data are provided as a Source Data file.

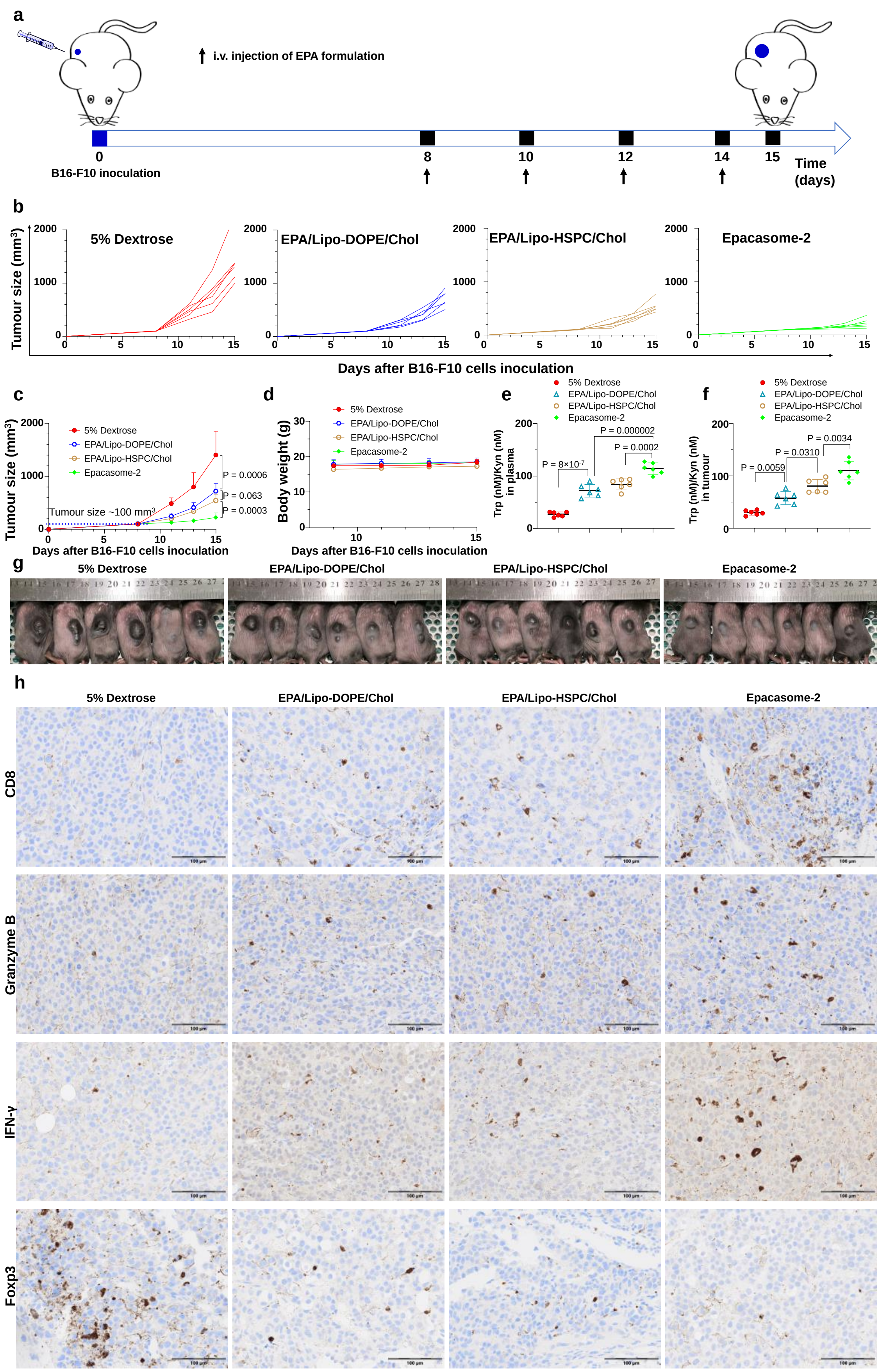

Supplementary Figure 15. a, Drug administration timeline scheme of antitumour efficacy in subcutaneous (s.c.) B16-F10 tumour model (n = 6 mice, tumours: ~100 mm^3), mice i.v.

injected with EPA/Lipo-DOPE/Chol, EPA/Lipo-HSPC/Chol, Epacasome-2 at eq. 41 mg EPA/kg on day 8, 10, 12 and 14 from day 8. **b**, Individual tumour growth curves. **c-d**, Average tumour size growth curves (**c**) and body weight (**d**). **e**,**f**, The ratio of Trp (nM)/kyn (nM) concentration in plasma (**e**) and tumours (**f**). **g**, Mice bearing s.c. B16-F10 tumour images taken on day 15. (**h**) Representative IHC staining of intratumoural CD8, Granzyme B, IFN-γ, and Foxp3 on day 15, scale bar = 100 μm, (n = 6 mice, similar results were observed). Data in **c-f** are expressed as mean ± s.d. (n = 6 mice). Statistical significance was determined by one-way ANOVA followed by Tukey's multiple comparisons test. Source data are provided as a Source Data file.

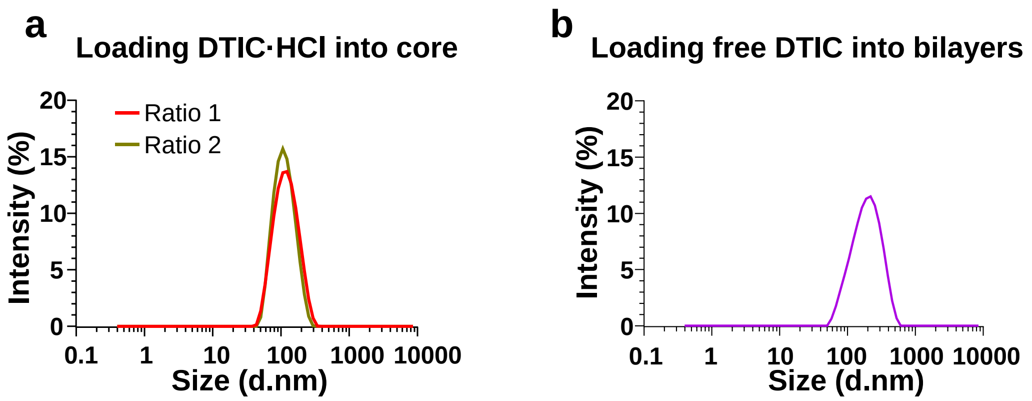

Supplementary Figure 16. The representative DLS size distribution by intensity for **DTIC·HCl** laden Epacasome-2 (**a**) and free DTIC laden Epacasome-2 (**b**) from **Fig. 5g**. Source data are provided as a Source Data file.

a

	SM (molar %)	Cholesterol (molar %)	DSPE-PEG$_{2K}$ (molar %)	DTIC input (mg/mL)	DTIC DLC (weight %)	DLS Size by intensity (d.nm)	Zeta Potential (mV)	PDI
**Loading DTIC·HCl into core**	82.82	12.78	4.40	95	15.86	125.0 ± 6.4	-19.3 ± 5.5	0.107 ± 0.048

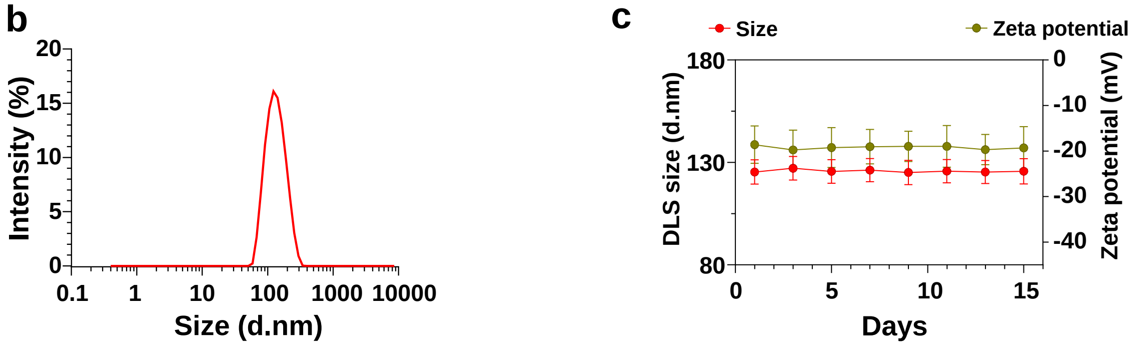

Supplementary Figure 17. Development and physicochemical characterizations of DTIC/Lipo-SM/Chol. **a,** A table shows the physicochemical characterizations of DTIC/Lipo-SM/Chol composed of SM/Cholesterol/DSPE-PEG$_{2K}$ with indicated molar ratios. **b,** The representative DLS size distribution by intensity for DTIC/Lipo-SM/Chol. **c**, DLS size and zeta potential of DTIC/Lipo-SM/Chol monitoring over a 15-day period at 4 °C. Data are represented as mean ± s.d. (n = 3 independent experiments). Source data are provided as a Source Data file.

EPA	DTIC/Lipo-SM/Chol + Epacasome-2	DTIC/Epacasome-2
**$T_{1/2}$ (h)**	4.09 ± 0.23	6.66 ± 0.65
**V ((µg)/(µg/ml))**	1.08 ± 0.02	1.01 ± 0.03
**CL(µg)/(µg/ml)/h)**	0.18 ± 0.01	0.11 ± 0.01
**AUC_{0-t} (µg/ml*h)**	4,397.82 ± 154.87	7,167.67 ± 708.02
**AUC_{0-inf} (µg/ml*h)**	4,475.79 ± 176.12	7,827.94 ± 954.96
**AUMC (µg/ml*h^2)**	26,464.43 ± 2,553.66	75,862.80 ± 16,994.51
**MRT (h)**	5.90 ± 0.33	9.62 ± 0.94
**V_{ss} (µg/(µg/ml))**	1.08 ± 0.02	1.01 ± 0.03

Supplementary Table 2. A table delineating various pharmacokinetic parameters of EPA from **Fig. 5l** (n = 3 mice). Source data are provided as a Source Data file.

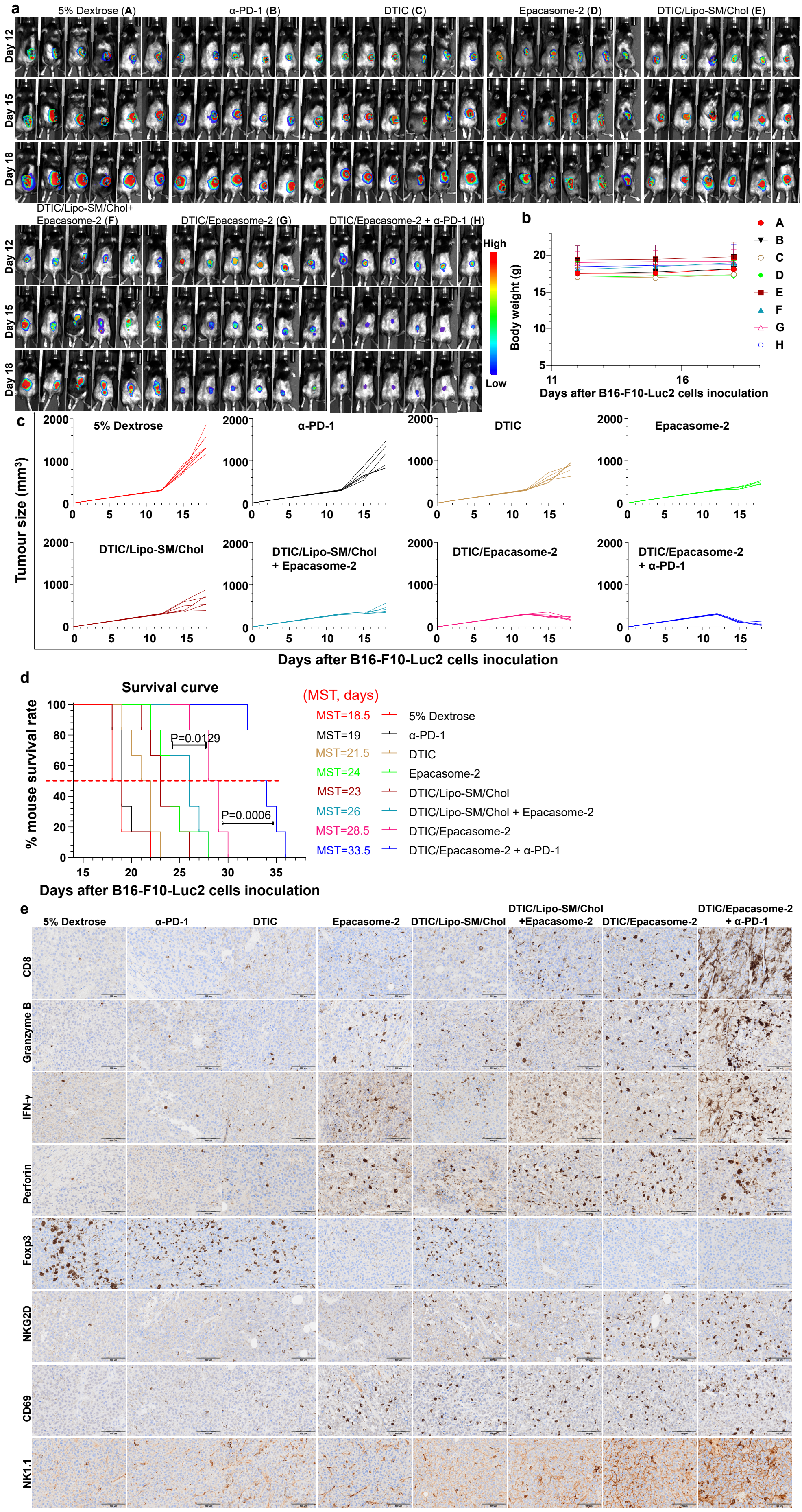
a
5% Dextrose (A)
α-PD-1 (B)
DTIC (C)
Epacasome-2 (D)
DTIC/Lipo-SM/Chol (E)
Day 12
Day 15
Day 18
DTIC/Lipo-SM/Chol+ Epacasome-2 (F)
DTIC/Epacasome-2 (G)
DTIC/Epacasome-2 + α-PD-1 (H)
High
Low
b
Body weight (g)
Days after B16-F10-Luc2 cells inoculation
c
Tumour size (mm³)
5% Dextrose
α-PD-1
DTIC
Epacasome-2
DTIC/Lipo-SM/Chol
DTIC/Lipo-SM/Chol + Epacasome-2
DTIC/Epacasome-2
DTIC/Epacasome-2 + α-PD-1
Days after B16-F10-Luc2 cells inoculation
d
Survival curve
% mouse survival rate
(MST, days)
MST=18.5 5% Dextrose
MST=19 α-PD-1
MST=21.5 DTIC
MST=24 Epacasome-2
MST=23 DTIC/Lipo-SM/Chol
MST=26 DTIC/Lipo-SM/Chol + Epacasome-2
MST=28.5 DTIC/Epacasome-2
MST=33.5 DTIC/Epacasome-2 + α-PD-1
P=0.0129
P=0.0006
Days after B16-F10-Luc2 cells inoculation
e
5% Dextrose
α-PD-1
DTIC
Epacasome-2
DTIC/Lipo-SM/Chol
DTIC/Lipo-SM/Chol +Epacasome-2
DTIC/Epacasome-2
DTIC/Epacasome-2 + α-PD-1
CD8
Granzyme B
IFN-γ
Perforin
Foxp3
NKG2D
CD69
NK1.1

Supplementary Figure 18. a, Mice Lago bioluminescence imaging (BLI) on day 12, 15 and 18. **b-c**, Average mouse body weight (**b**) and individual tumour growth curves (**c**) and in therapeutic efficacy study presented in **Fig. 6**. **d**, Kaplan-Meier survival curves from an independent efficacy study in B16-F10 tumour mice (n = 6 mice, tumours: ~300 mm^3) after receiving the same treatment as **Fig. 6**. (**e**) Representative IHC staining of intratumoural CD8, Granzyme B, IFN-γ, perforin, Foxp3, NKG2D, CD69 and NK1.1 on day 18, scale bar = 100 μm (n = 6 mice, similar results were observed). Data in **b** are represented as mean ± s.d. (n = 6 mice), survival curves were compared using the log-rank Mantel–Cox test. Source data are provided as a Source Data file.

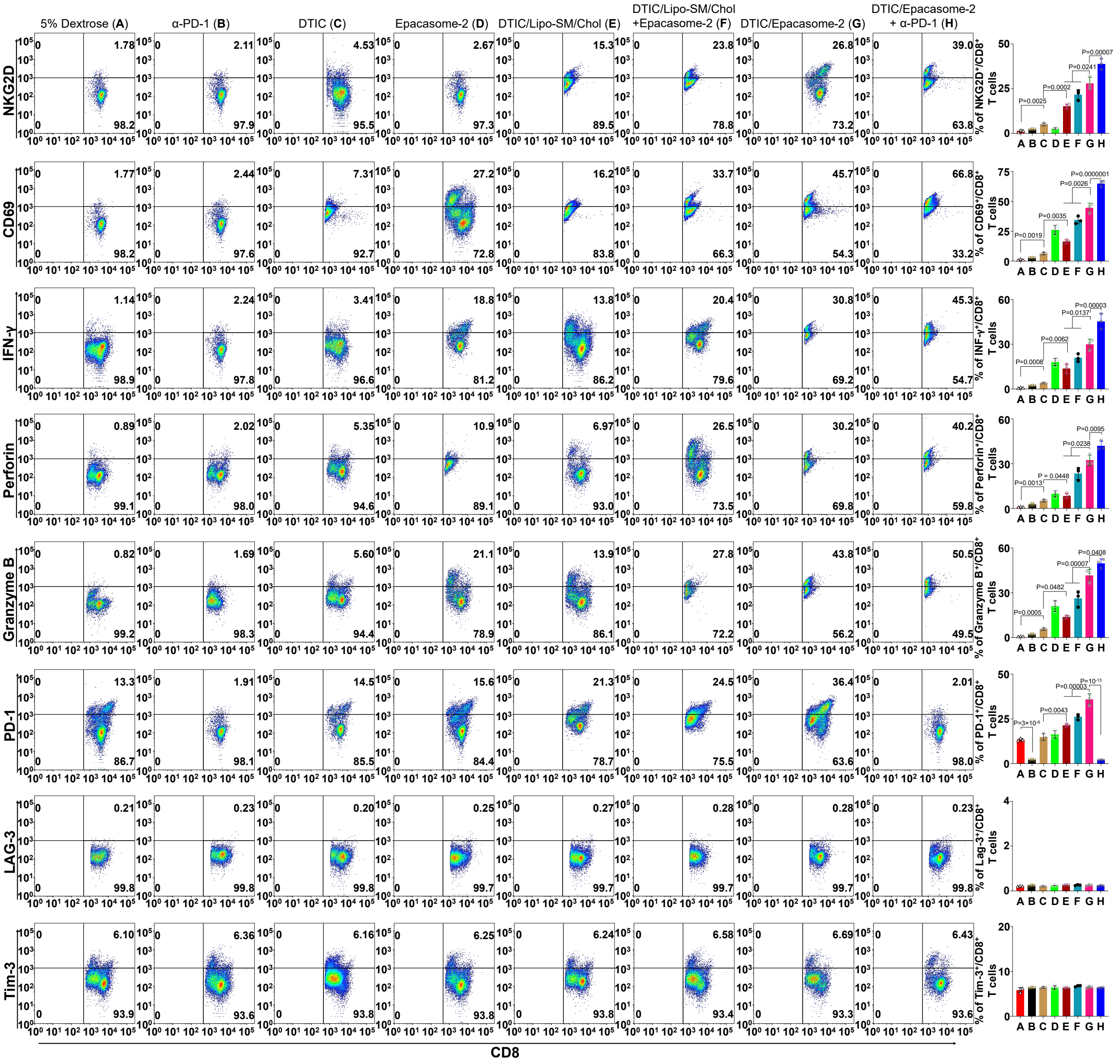

Supplementary Figure 19. Representative flow cytometric plots of intratumoural NKG2D$^+$, CD69$^+$, IFN-γ$^+$, Perforin$^+$, Granzyme B$^+$, PD-1$^+$, Lag-3$^+$ and Tim-3$^+$ associated CD8$^+$ T cells and their respective quantification (right panel, 3 tumours were randomly chosen from an independent assay on day 18 after receiving the same treatment as **Fig. 6a**). The flow cytometric gating strategies are placed in **Supplementary Figure 22**. Data are expressed as mean ± s.d. Statistical significance was determined by one-way ANOVA followed by Tukey's multiple comparisons. Source data are provided as a Source Data file.

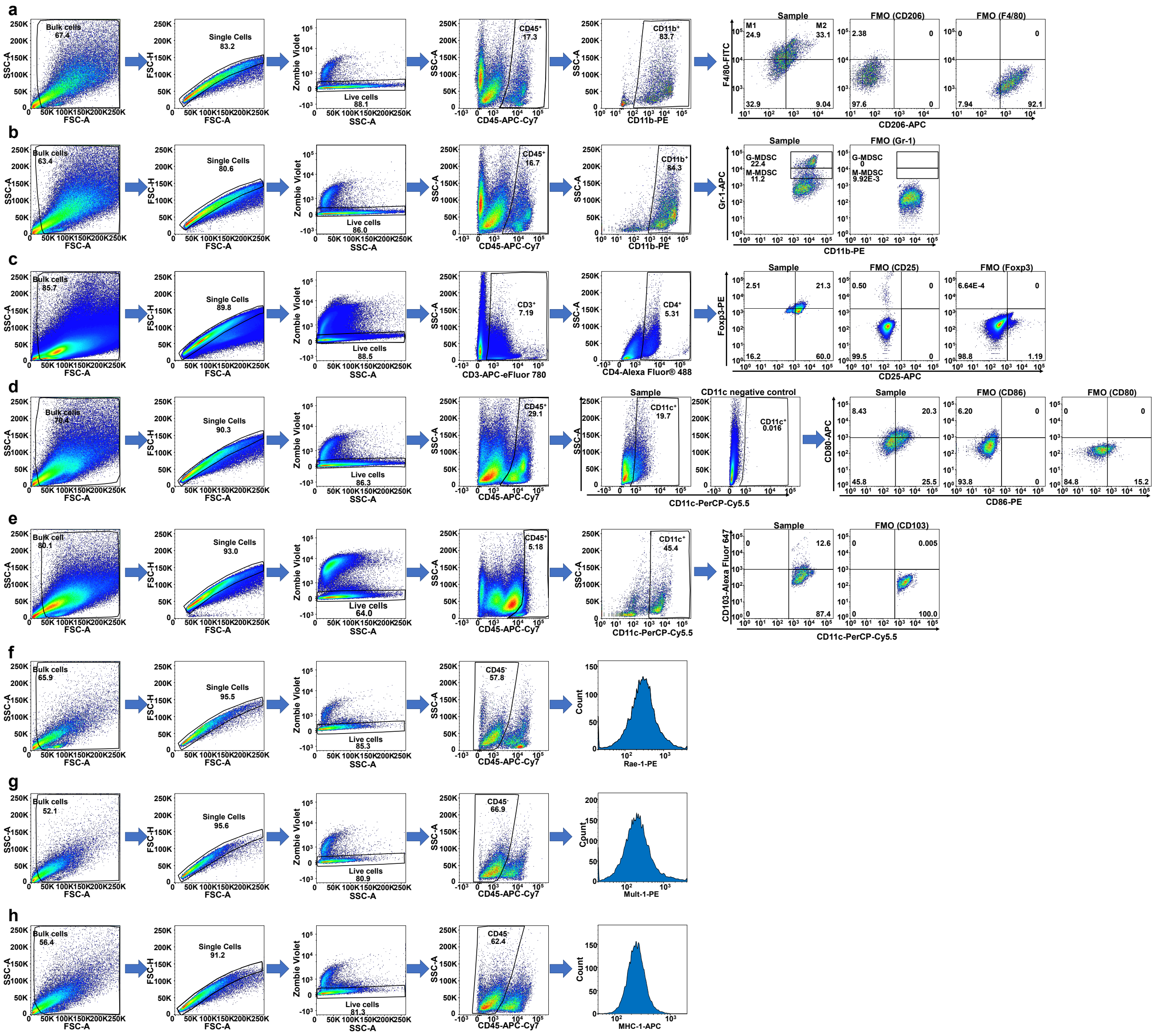

Supplementary Figure 20. Gating strategy used to define intratumoural ($CD45^+/CD11b^+/F4/80^+/CD206^{\pm}$) TAMs cells (**a**), ($CD45^+/CD11b^+/Gr\text{-}1^+$) MDSC cells (**b**), ($CD4^+/CD3^+/CD25^+/Foxp3^+$) Tregs (**c**), $CD45^+/CD11c^+/CD80^+/CD86^+$ (**d**), $CD45^+/CD11c^+/CD103^+$ (**e**) DCs in **Supplementary Figure 12b**, and $CD45^-/Rae\text{-}1^+$ (**f**), $CD45^-/Mult\text{-}1^+$ (**g**), $CD45^-/MHC\text{-}I^+$ (**h**) tumour cells in **Fig. 6h-m**.

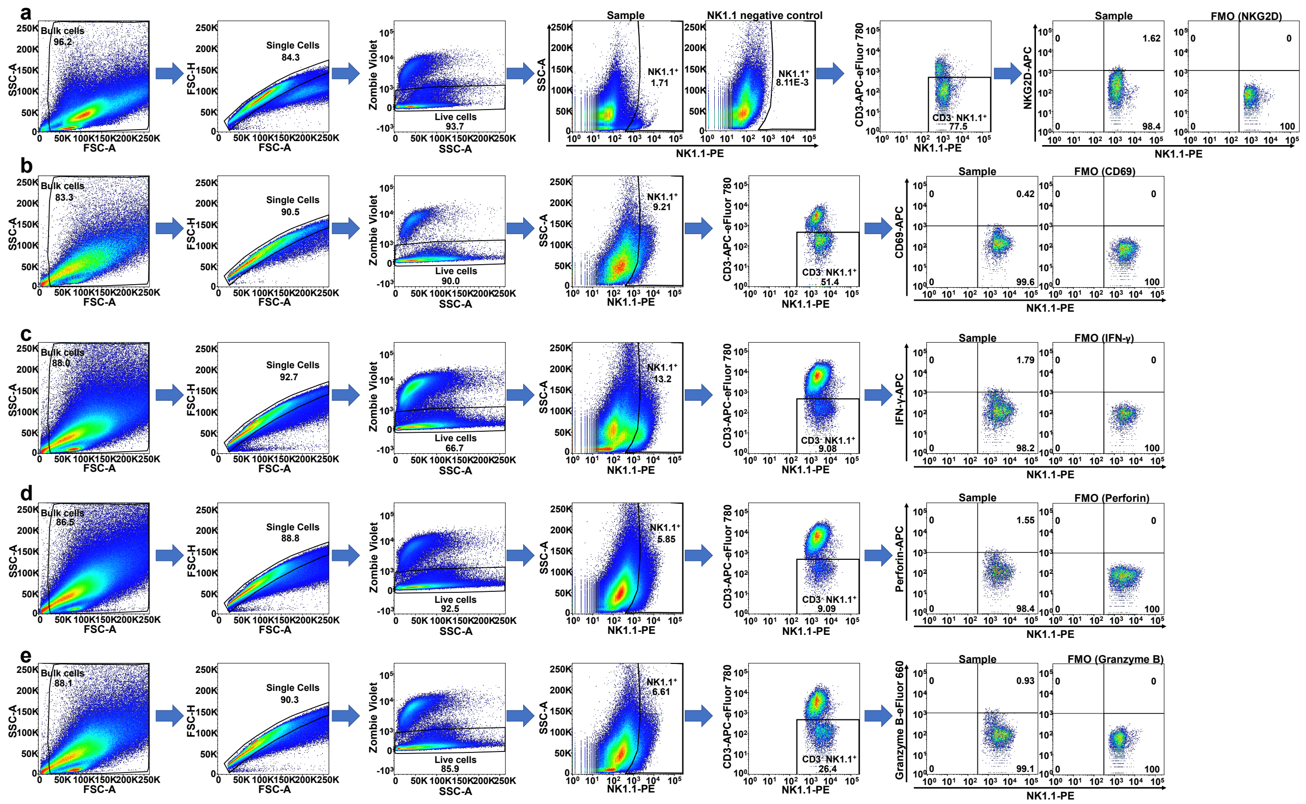

Supplementary Figure 21. Gating strategy used to define intratumoural CD3⁻/NK1.1⁺/NKG2D⁺ (**a**), CD3⁻/NK1.1⁺/CD69⁺ (**b**), CD3⁻/NK1.1⁺/IFN-γ⁺ (**c**), CD3⁻/NK1.1⁺/perforin⁺ (**d**) and CD3⁻/NK1.1⁺/granzyme B⁺ (**e**) NK cells in **Fig. 6n**.

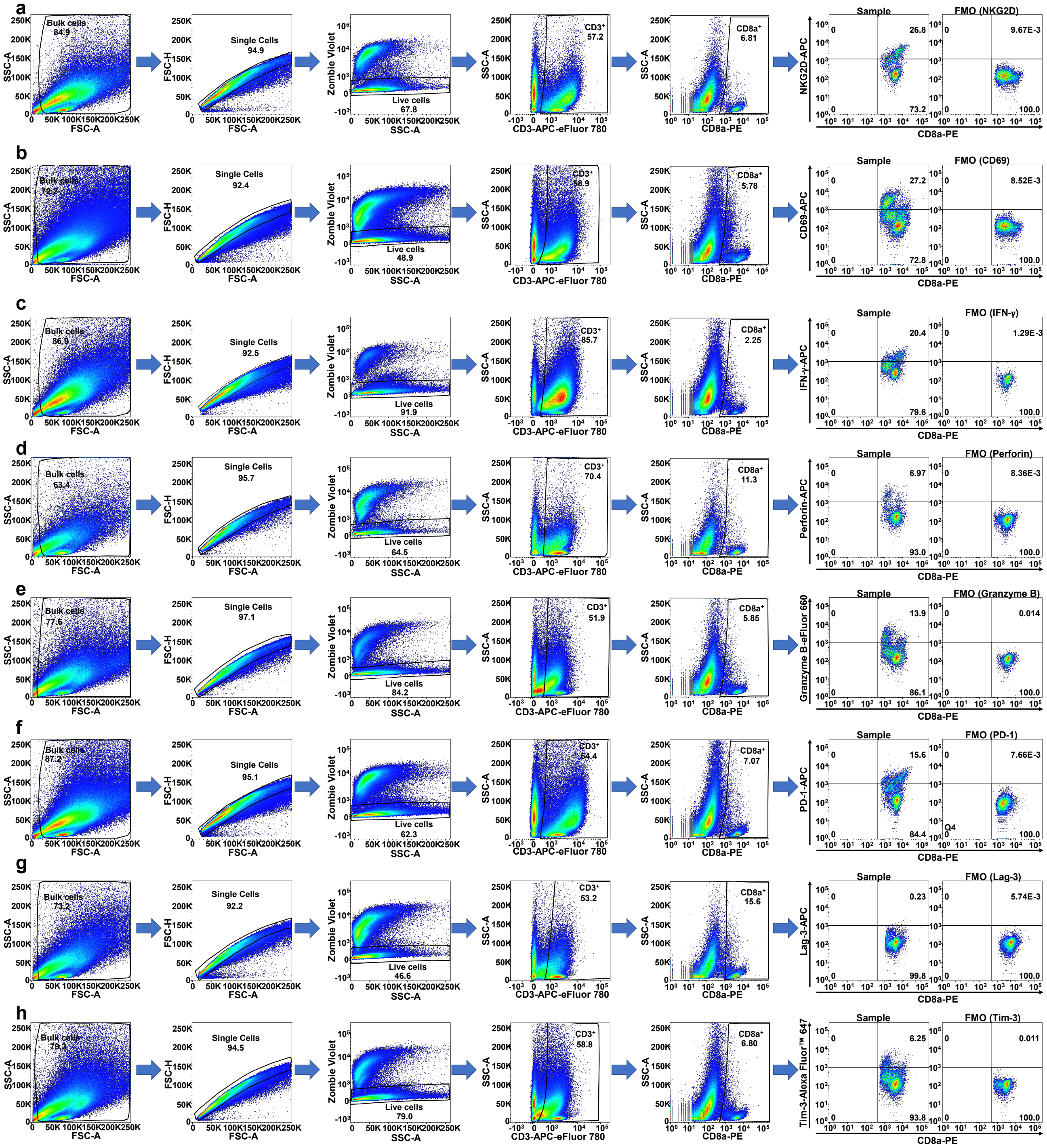

Supplementary Figure 22. Gating strategy used to define intratumoural $CD3^+/CD8^+/NKG2D^+$ (**a**), $CD3^+/CD8^+/CD69^+$ (**b**), $CD3^+/CD8^+$/IFN-γ^+ (**c**), $CD3^+/CD8^+$/perforin$^+$ (**d**), $CD3^+/CD8^+$/granzyme B^+ (**e**), $CD3^+/CD8^+$/PD-1^+ (**f**), $CD3^+/CD8^+$/Lag-3^+ (**g**) and $CD3^+/CD8^+$/Tim-3^+ (**h**) T cells in **Supplementary Figure 12b** and **19**.

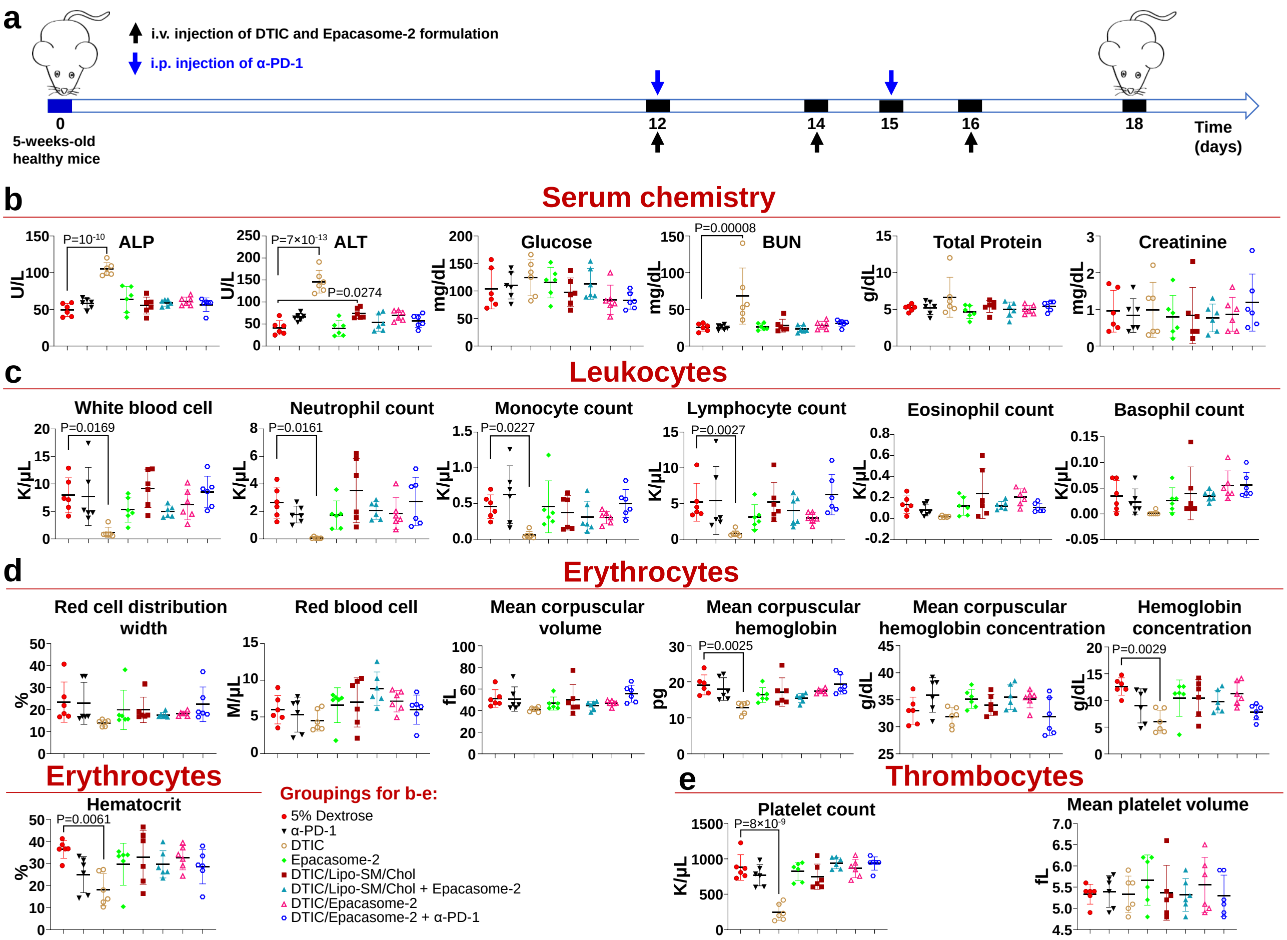

Supplementary Figure 23. a, Drug administration timeline scheme in healthy C57BL/6 mice that received the same treatments as **Fig. 6a**. **b-e**, On day 18, blood was withdrawn for comprehensive serum chemistry (**b**), leukocytes (**c**), erythrocytes (**d**) and thrombocytes (**e**) analysis. Data are expressed as mean ± s.d. (n = 6 mice). Statistical significance was determined by one-way ANOVA followed by Tukey's multiple comparisons test. Source data are provided as a Source Data file.

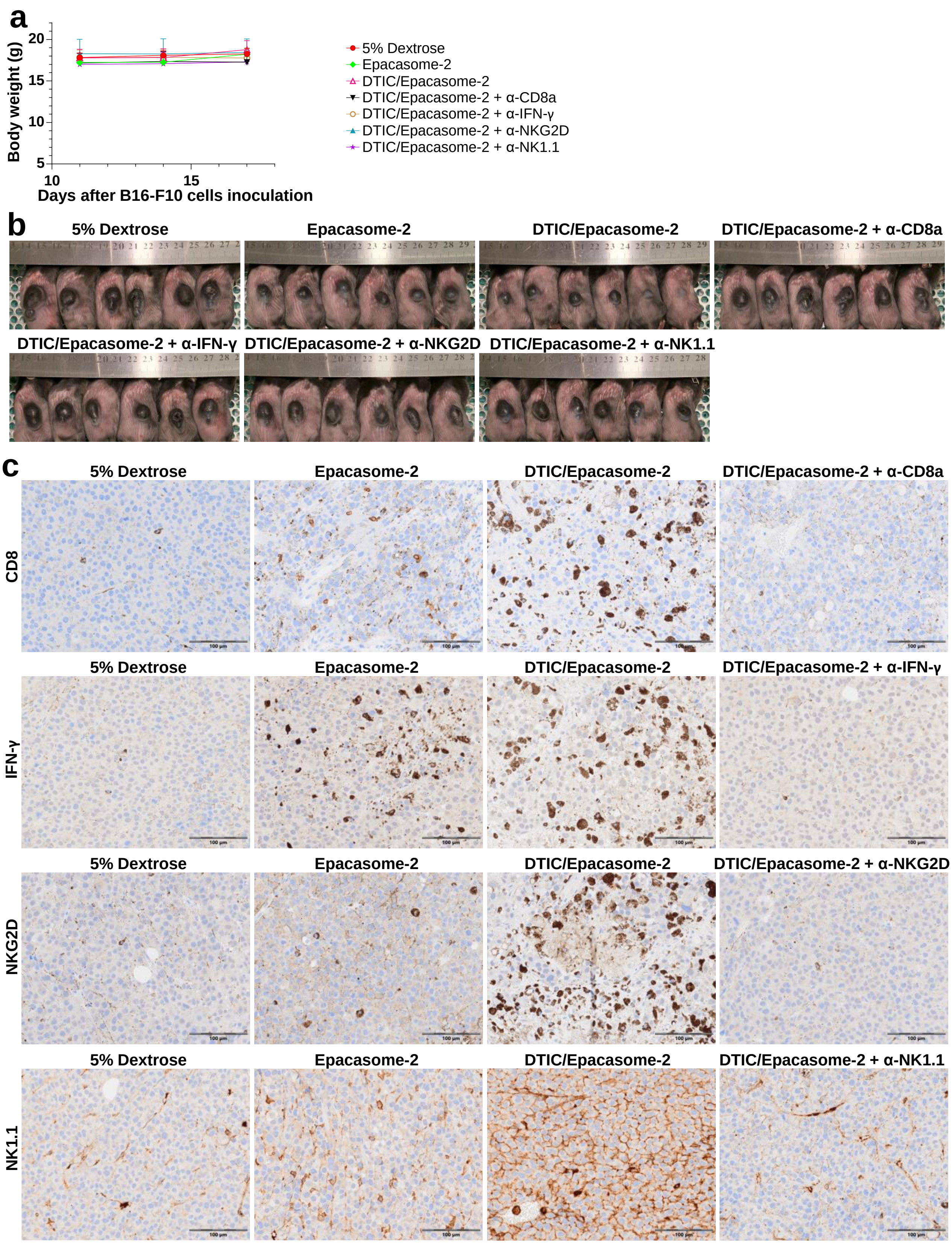

Supplementary Figure 24. Mouse body weight (**a**) and mice bearing s.c. B16-F10 tumour images taken on day 17 (**b**) in therapeutic efficacy study presented in **Fig. 7** (n = 6 mice). (**c**) Representative IHC staining of intratumoural CD8, IFN-γ, NKG2D and NK1.1 on day 17 from an independent study after receiving the same treatment as **Fig. 7**, scale bar = 100

μm (n = 6 mice, similar results were observed). Data in **a** is represented as mean ± s.d. (n = 6 mice). Source data are provided as a Source Data file.

a

Group	Drug administration sequence								
	Day 11	Day 12	Day 13	Day 14	Day 15	Day 16	Day 17	Day 18	Day 19
A	5% dextrose	5% dextrose	5% dextrose	5% dextrose	5% dextrose	5% dextrose	5% dextrose	5% dextrose	5% dextrose
B	α-PD-1	Epacasome-2	DTIC	α-PD-1	Epacasome-2	DTIC	α-PD-1	Epacasome-2	DTIC
C	α-PD-1	DTIC	Epacasome-2	α-PD-1	DTIC	Epacasome-2	α-PD-1	DTIC	Epacasome-2
D	Epacasome-2	DTIC	α-PD-1	Epacasome-2	DTIC	α-PD-1	Epacasome-2	DTIC	α-PD-1
E	Epacasome-2	α-PD-1	DTIC	Epacasome-2	α-PD-1	DTIC	Epacasome-2	α-PD-1	DTIC
F	DTIC	Epacasome-2	α-PD-1	DTIC	Epacasome-2	α-PD-1	DTIC	Epacasome-2	α-PD-1
G	DTIC	α-PD-1	Epacasome-2	DTIC	α-PD-1	Epacasome-2	DTIC	α-PD-1	Epacasome-2
H	DTIC, Epacasome-2, α-PD-1	NA	NA	DTIC, Epacasome-2, α-PD-1	NA	NA	DTIC, Epacasome-2, α-PD-1	NA	NA

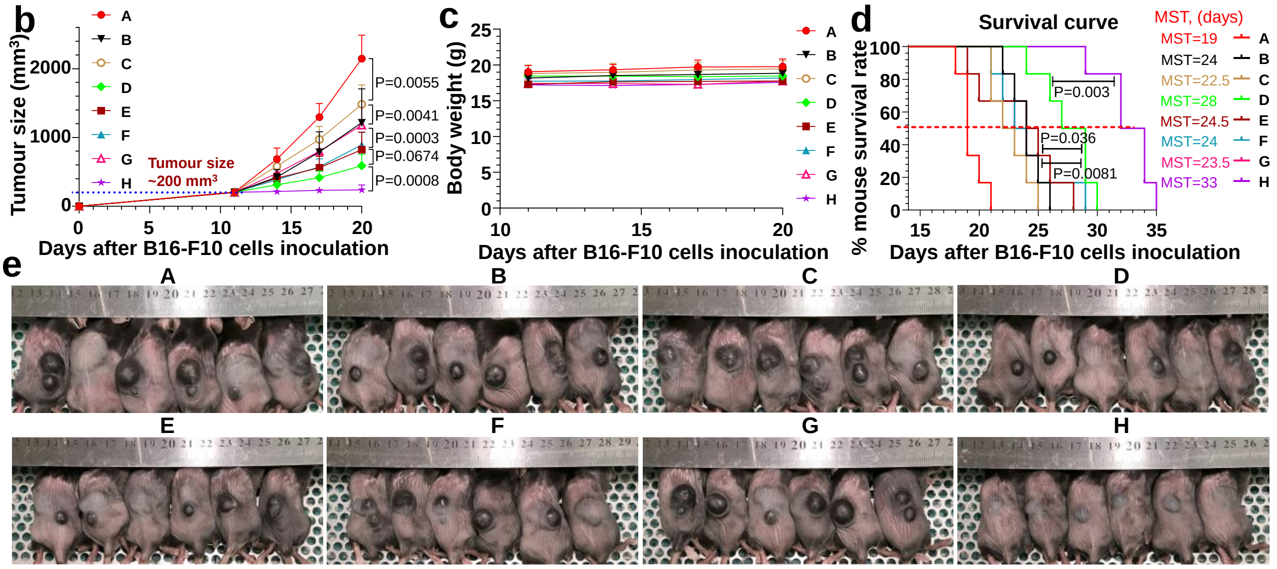

Supplementary Figure 25. a, Drug administration table scheme of antitumour efficacy in subcutaneous (s.c.) B16-F10 tumour model (n = 6 mice, tumours: ~200 mm^3), mice injected with Epacasome-2 (i.v. at eq. 41 mg EPA/kg), DTIC (i.v. 75 mg/kg) and combined with i.p. α-PD-1 (BioXCell, clone RMP1-14, 100 μg per mouse) from day 11. **b-c**, Average tumour size growth curves (**b**) and body weight (**c**). **d**, Kaplan-Meier survival curves. **e**, Mice bearing s.c. B16-F10 tumour images taken on day 20. Data in **b-c** are expressed as mean ± s.d. (n = 6 mice) Statistical significance was determined by one-way ANOVA followed by Tukey's multiple comparisons test; survival curves were compared using the log-rank Mantel–Cox test. Source data are provided as a Source Data file.

Group (n = 5 mice)	Median survival time (MST, days)	Time to reach endpoint (TTE, days)					Tumour growth delay (TGD, days)					Increased live span (ILS, %)				
5% Dextrose	22	21	24	23	21	22	NA	NA	NA	NA	NA	NA	NA	NA	NA	NA
α-PD-1	23	24	22	23	24	23	3.2	1.8	0.7	1	2.4	9.1%	0.0%	4.5%	9.1%	4.5%
DTIC/Lipo-SM/Chol + Epacasome-2	27	24	26	27	28	29	6.2	3	4.2	2.3	2.7	27.3%	18.2%	31.8%	9.1%	22.7%
DTIC/Epacasome-2	30	28	29	30	31	32	8	6.9	6.7	6.5	5.6	45.5%	40.9%	36.4%	31.8%	27.3%
DTIC/Lipo-SM/Chol + Epacasome-2 + α-PD-1	31	27	28	32	31	33	8	7.5	9.3	8.9	6.5	40.9%	27.3%	50.0%	45.5%	22.7%
DTIC/Epacasome-2 + α-PD-1	35	35	36	37	32	35	14	12.8	11.6	15.8	12.2	63.6%	59.1%	45.5%	68.2%	59.1%
**Statistical significance comparison**		P value by one-way ANOVA followed by Tukey's multiple comparisons test														
DTIC/Lipo-SM/Chol + Epacasome-2 vs DTIC/Epacasome-2		0.0817					0.0194					0.0608				
DTIC/Lipo-SM/Chol + Epacasome-2 + α-PD-1 vs DTIC/Epacasome-2 + α-PD-1		0.0031					0.00004					0.0018				
DTIC/Epacasome-2 vs DTIC/Epacasome-2 + α-PD-1		0.0020					0.000001					0.0012				

Supplementary Table 3. A table shows the median survival time (MST), time to reach endpoint (TTE), tumour growth delay (TGD) and increased live span (ILS) from **Fig. 8** (n = 5 mice). Statistical significance was determined by one-way ANOVA followed by Tukey's multiple comparisons test. Source data are provided as a Source Data file.

Species	Dose in clinic	Dose in mg/kg	To convert human dose in mg/kg to mouse equivalent dose in mg/kg, either:	
			Divide animal dose by	Multiply animal dose by
Human (60 kg)	100 mg EPA twice daily (Bid.)	3.33 mg/kg		
Mouse		41 mg/kg	12.3	0.08

Supplementary Table 4. Conversion the human dose of EPA to mouse equivalent dose.

Supplementary References

1 Tahaghoghi-Hajghorbani, S., Khoshkhabar, R., Rafiei, A., Ajami, A., Nikpoor, A. R., Jaafari, M. R. & Badiee, A. Development of a novel formulation method to prepare liposomal Epacadostat. *European journal of pharmaceutical sciences : official journal of the European Federation for Pharmaceutical Sciences* **165**, 105954 (2021).

2 Chen, Y., Du, Q., Zou, Y., Guo, Q., Huang, J., Tao, L., Shen, X. & Peng, J. Co-delivery of doxorubicin and epacadostat via heparin coated pH-sensitive liposomes to suppress the lung metastasis of melanoma. *International journal of pharmaceutics* **584**, 119446 (2020).

3 Chen, Y., Xia, R., Huang, Y., Zhao, W., Li, J., Zhang, X., Wang, P., Venkataramanan, R., Fan, J., Xie, W., Ma, X., Lu, B. & Li, S. An immunostimulatory dual-functional nanocarrier that improves cancer immunochemotherapy. *Nature communications* **7**, 13443 (2016).

4 Chen, Y., Huang, Y., Li, Q., Luo, Z., Zhang, Z., Huang, H., Sun, J., Zhang, L., Sun, R., Bain, D. J., Conway, J. F., Lu, B. & Li, S. Targeting Xkr8 via nanoparticle-mediated in situ co-delivery of siRNA and chemotherapy drugs for cancer immunochemotherapy. *Nature nanotechnology* **18**, 193-204 (2023).
