# Supplementary material for: Sphingomyelin-derived nanovesicles for the delivery of the IDO1 inhibitor epacadostat enhance metastatic and post-surgical melanoma immunotherapy
Source: Nat Commun. 2023 Nov 9;14:7235. doi: 10.1038/s41467-023-43079-4 (PMC10636136; doi:10.1038/s41467-023-43079-4)
Supplement: Supplementary file 3 — Reporting Summary [file 41467_2023_43079_MOESM3_ESM.pdf]

nature portfolio

Corresponding author(s): Jianqin Lu

Last updated by author(s): Oct 26, 2023

Reporting Summary

Nature Portfolio wishes to improve the reproducibility of the work that we publish. This form provides structure for consistency and transparency in reporting. For further information on Nature Portfolio policies, see our Editorial Policies and the Editorial Policy Checklist.

Statistics

For all statistical analyses, confirm that the following items are present in the figure legend, table legend, main text, or Methods section.

n/a	Confirmed	
☐	☒	The exact sample size (n) for each experimental group/condition, given as a discrete number and unit of measurement
☐	☒	A statement on whether measurements were taken from distinct samples or whether the same sample was measured repeatedly
☐	☒	The statistical test(s) used AND whether they are one- or two-sided<br>*Only common tests should be described solely by name; describe more complex techniques in the Methods section.*
☐	☒	A description of all covariates tested
☐	☒	A description of any assumptions or corrections, such as tests of normality and adjustment for multiple comparisons
☐	☒	A full description of the statistical parameters including central tendency (e.g. means) or other basic estimates (e.g. regression coefficient) AND variation (e.g. standard deviation) or associated estimates of uncertainty (e.g. confidence intervals)
☐	☒	For null hypothesis testing, the test statistic (e.g. F, t, r) with confidence intervals, effect sizes, degrees of freedom and P value noted<br>*Give P values as exact values whenever suitable.*
☐	☒	For Bayesian analysis, information on the choice of priors and Markov chain Monte Carlo settings
☒	☐	For hierarchical and complex designs, identification of the appropriate level for tests and full reporting of outcomes
☒	☐	Estimates of effect sizes (e.g. Cohen's d, Pearson's r), indicating how they were calculated

Our web collection on statistics for biologists contains articles on many of the points above.

Software and code

Policy information about availability of computer code

Data collection	NMR spectra were acquired by Bruker topspin software (v. 2.1). HPLC spectra were acquired by ChemStation Rev.A. software (v. 10.01, Agilent Technology). DLS size and zeta potential data were acquired by Zetasizer software (v. 7.13). Cryo-EM image were acquired by Tecnai User Interface software (v. 3.1.5) and EMMenu. Ultraviolet absorption and fluorescence were acquired by SoftMax® Pro (v. 7.1.0). Serum chemistry and hematological counts were acquired by Liasys 330 and Hemavet 950FS, respectively. Immunofluorescence images were acquired by Zeiss LSM880 inverted confocal microscope (Zen Black software (v. 14.022.021)). Pharmacokinetic parameters were acquired by PKSolver software (version 2.0). In vivo bioluminescence and fluorescence images were acquired by Aura 64 Bit Analysis software (v. 3.2.0). Flow cytometry data were acquired by BD FACSCanto II (BD FACSDiva Software, version 8.01). Histology images were acquired by Olympus VS200 slide scanner (version OlyVIA V4.1).
Data analysis	NMR data spectra were analyzed by MestReNova (v. 6.0.2). HPLC data and spectra were analyzed by ChemStation Rev.A. software (v. 10.01, Agilent Technology). In vivo bioluminescence and fluorescence images were analysed by Aura 64 Bit Analysis software (v. 3.2.0). All statistical analyses were performed with Graphpad Prism 8. Flow cytometry were analyzed by FlowJo software (version 10.0.7, TreeStar)

For manuscripts utilizing custom algorithms or software that are central to the research but not yet described in published literature, software must be made available to editors and reviewers. We strongly encourage code deposition in a community repository (e.g. GitHub). See the Nature Portfolio guidelines for submitting code & software for further information.

Data

Policy information about availability of data

All manuscripts must include a data availability statement. This statement should provide the following information, where applicable:

- Accession codes, unique identifiers, or web links for publicly available datasets
- A description of any restrictions on data availability
- For clinical datasets or third party data, please ensure that the statement adheres to our policy

All the data supporting the findings of this study are available within the article and its Supplementary Information. Source data are provided with this paper.

Research involving human participants, their data, or biological material

Policy information about studies with human participants or human data. See also policy information about sex, gender (identity/presentation), and sexual orientation and race, ethnicity and racism.

Reporting on sex and gender	N/A
Reporting on race, ethnicity, or other socially relevant groupings	N/A
Population characteristics	N/A
Recruitment	N/A
Ethics oversight	N/A

Note that full information on the approval of the study protocol must also be provided in the manuscript.

Field-specific reporting

Please select the one below that is the best fit for your research. If you are not sure, read the appropriate sections before making your selection.

☒ Life sciences ☐ Behavioural & social sciences ☐ Ecological, evolutionary & environmental sciences

For a reference copy of the document with all sections, see nature.com/documents/nr-reporting-summary-flat.pdf

Life sciences study design

All studies must disclose on these points even when the disclosure is negative.

Sample size	The sample sizes were determined by the published literature (Nat. Nanotechnol. 2021,16(10):1130-1140)
Data exclusions	No data were excluded.
Replication	Most in vitro experiments were have 3 biologically independent samples or repeated independently for at least 3 times. All in vivo studies were repeated at least 5-6 mice per group. The detailed information is also described in the figure legends and methods section.
Randomization	The experimental groups were allocated randomly.
Blinding	No formal blinding was used. The serum chemistry, hematological counts, Cryo-EM was conducted by independent scientists, who were unaware of the treatment conditions, in respective core facilities. For other assays, the investigators were not blinded to the group allocation because the data analyses were based on objectively measurable data.

Reporting for specific materials, systems and methods

We require information from authors about some types of materials, experimental systems and methods used in many studies. Here, indicate whether each material, system or method listed is relevant to your study. If you are not sure if a list item applies to your research, read the appropriate section before selecting a response.

Materials & experimental systems

n/a	Involved in the study
	☒ Antibodies
	☒ Eukaryotic cell lines
☒	Palaeontology and archaeology
	☒ Animals and other organisms
☒	Clinical data
☒	Dual use research of concern
☒	Plants

Methods

n/a	Involved in the study
☒	ChIP-seq
	☒ Flow cytometry
☒	MRI-based neuroimaging

Antibodies

Antibodies used

Flow cytometry Experiments:
Anti-CD45-APC-Cy™7 (BD Biosceinces, Cat#: 557659, Rat Anti-Mouse (30-F11), dilution: 1/100),
Anti-CD8a-PE (BD Bioseinces, Cat#: 561095, Hamster Anti-Mouse (53-6.7), dilution: 1/100),
Anti-CD11c-PerCP-Cy5.5 (BD Bioseinces, Cat#: 560584, Hamster Anti-Mouse (HL3), dilution: 1/100),
Anti-CD80-APC (BD Bioseinces, Cat#: 553766, Hamster Anti-Mouse (16-10A1), dilution: 1/100),
Anti-CD86-PE (BD Bioseinces, Cat#: 553692, Rat Anti-Mouse (GL1), dilution: 1/100),
Anti-LAG-3(CD223)-APC (BD Bioseinces, Cat#: #562346, Rat Anti-Mouse (C9B7W), dilution: 1/100)
Anti-CD3-APC-eFluor 780 (eBioscience, Cat#: 47-0032-82, Rat Anti-Mouse (17A2), dilution: 1/100),
Anti-CD25-APC (eBioscience, Cat#: 17-0251-82, Rat Anti-Mouse (PC61.5), dilution: 1/100),
Anti-Foxp3-PE (eBioscience, Cat#: 563101, Rat Anti-Mouse (R16-715), dilution: 1/100),
Anti-Mult-1 (eBioscience, Cat#: 5013229, Hamster Anti-Mouse (5D10), dilution: 1/100),
Secondary antibody-PE (eBioscience, Cat#: 5010771, goat anti-Armenian Hamster IgG (H+L); 1 µg/sample),
Anti-Rae-1-PE (Bio-Techne, Cat#: FAB17582P, Rat Anti-Mouse (186107), dilution: 1/100),
Anti-MHC class I-Alexa Fluor® 647 (BioLegend, Cat#: 116512, Anti-Mouse (AF6-88.5), dilution: 1/100),
Anti-Granzyme B-eFluor 660 (eBioscience, Cat#: 50-8898-82, Rat Anti-Mouse (NGZB), dilution: 1/100),
Anti-CD4-Alexa Fluor 488 (BioLegend, Cat#: 100529, Rat Anti-Mouse (RM4-5), dilution: 1/100),
Anti-perforin-APC (BioLegend, Cat#: 154304, Rat Anti-Mouse (S16009A), dilution: 1/100),
Anti-NKG2D-APC (BioLegend, Cat#: 130212, Rat Anti-Mouse (CX5), dilution: 1/100),
Anti-CD69-APC (BioLegend, Cat#: 104514, Hamster Anti-Mouse (H1.2F3), dilution: 1/100),
Anti-Gr-1-APC (BioLegend, Cat#: 108412, Rat Anti-Mouse (RB6-8C5), dilution: 1/100),
Anti-CD206-APC (BioLegend, Cat#: 141708, Rat Anti-Mouse (C068C2), dilution: 1/100),
Anti-F4/80-FITC (BioLegend, Cat#: 123108, Rat Anti-Mouse (BM8), dilution: 1/100),
Anti-CD11b-PE (BioLegend, Cat#: 101208, Rat Anti-Mouse (M1/70), dilution: 1/100),
Anti-NK1.1-PE (BioLegend, Cat#: 156504, Anti-Mouse (S17016D), dilution: 1/100),
Anti-IFN-γ-APC (BioLegend, Cat#: 505810, Rat Anti-Mouse (XMG1.2), dilution: 1/100),
Anti-PD-1 (CD279)-APC (BioLegend, Cat#: 135210, Rat Anti-Mouse (29F.1A12), dilution: 1/100)
Anti-Tim-3-Alexa Fluor® 647 (BioLegend, Cat#: 119744, Rat Anti-Mouse (RMT3-23), dilution: 1/100)

Immunohistochemistry Experiments:
Anti-CD8α (Abcam, Cat#: ab209775, Rabbit monoclonal [EPR20305] to CD8 alpha, dilution: 1/100),
Anti-granzyme B (Abcam, Cat#: ab4059, Rabbit polyclonal to Granzyme B, dilution: 1/100),
Anti-perforin (Abcam, Cat#: ab16074, Rat monoclonal [CB5.4] to Perforin, dilution: 1/600),
Anti-interferon gamma (Abcam, Cat#: ab9657, Rabbit polyclonal to Interferon gamma, dilution: 1/200),
Anti-NK1.1 (Invitrogen, Cat#: MA1-70100, mouse monoclonal to NK1.1, dilution: 1/100),
Anti-CD69 (Invitrogen, Cat#: PA5-102562, Rabbit polyclonal to CD69, dilution: 1/100)
Anti-NKG2D (Bioss, Cat#: BS-0938R, Rabbit polyclonal to NKG2D, dilution: 1/100)
Anti-Foxp3 (Cell Signaling, Cat#: 12653S, Rabbit monoclonal to mouse Foxp 3 (D6O8R), dilution: 1/100).

Immunofluorescence Experiments:
Anti-CD31 (a.k.a. PECAM-1) antibody (Abcam, Cat#: ab28364, Rabbit polyclonal to CD31, dilution: 1/50)
Alexa Fluor 488-conjugated secondary antibody (Abcam, Cat#: ab150073, Donkey polyclonal Secondary Antibody to Rabbit IgG - H&L dilution: 1/400)

Validation

All antibodies were verified by the supplier's websites and/or results presented in the manuscript.
The detailed information are listed as below:
Flow cytometry Experiments:

1) Anti-CD45-APC-Cy™7; Rat; application for Flow cytometry (Routinely Tested); reacts with mouse (QC Testing)
2) Anti-CD8a-PE; Hamster; application for Flow cytometry (Routinely Tested); reacts with mouse (QC Testing)
3) Anti-CD11c-PerCP-Cy5.5; Hamster; application for Flow cytometry (Routinely Tested), Immunofluorescence (Tested During

Development); reacts with mouse (QC Testing)
4) Anti-CD80-APC; Hamster; application for Flow cytometry (Routinely Tested), Blocking, Immunohistochemistry-frozen, Immunoprecipitation, In vivo exacerbation (Reported); reacts with Mouse (QC Testing), Dog (Reported)
5) Anti-CD86-PE; Rat; application for Flow cytometry (Routinely Tested); reacts with mouse (QC Testing)
6) Anti-LAG-3(CD223)-APC, Rat; application for Flow cytometry (Routinely Tested); reacts with mouse (QC Testing)
7) Anti-CD3-APC-eFluor 780; Rat; application for Flow cytometry; reacts with mouse
8) Anti-CD25-APC; Rat; application for Flow cytometry; reacts with mouse
9) Anti-Foxp3-PE; Rat; application for Intracellular staining Flow cytometry; reacts with mouse
10) Anti-Mult-1; Hamster; application for Intracellular staining Flow cytometry; reacts with mouse
11) Secondary antibody-PE; Goat; application for Intracellular staining Flow cytometry; reacts with Hamster
12) Anti-Rae-1-PE; Rat; application for Flow cytometry; reacts with mouse
13) Anti-MHC class I-Alexa Fluor® 647; Mouse, application for Flow cytometry; reacts with mouse
14) Anti-Granzyme B-eFluor 660; Rat; application for Flow cytometry; reacts with mouse
15) Anti-CD4-Alexa Fluor 488; Rat; application for Flow cytometry; reacts with mouse
16) Anti-perforin-APC; Rat; application for Flow cytometry; reacts with mouse
17) Anti-NKG2D-APC; Rat; application for Flow cytometry; reacts with mouse
18) Anti-CD69-APC; Hamster; application for Flow cytometry; reacts with mouse
19) Anti-Gr-1-APC; Rat; application for Flow cytometry; reacts with mouse
20) Anti-CD206-APC; Rat; application for Flow cytometry; reacts with mouse
21) Anti-F4/80-FITC; Rat; application for Flow cytometry; reacts with mouse
22) Anti-CD11b-PE; Rat; application for Flow cytometry; reacts with mouse
23) Anti-NK1.1-PE; Mouse; application for Flow cytometry; reacts with mouse
24) Anti-IFN-γ-APC; Rat; application for Flow cytometry; reacts with mouse
25) Anti-PD-1 (CD279)-APC; Rat; application for Flow cytometry; reacts with mouse
26) Anti-Tim-3-Alexa Fluor® 647; Rat; application for Flow cytometry; reacts with mouse

Immunohistochemistry Experiments:
1) Anti-CD8α; rabbit; suitable for IHC-P, WB; reacts with mouse
2) Anti-Interferon gamma antibody; rabbit; suitable for Sandwich enzyme-linked immunosorbent assay ELISA, (western blot) WB, Immunohistochemistry (IHC-P); reacts with human, recombinant fragment
3) Anti-granzyme B; rabbit; suitable for IHC-P, reacts with human
4) Anti-perforin; rat; reacts with mouse
5) Anti-NK1.1; mouse; suitable for IHC, react with human, mouse
6) Anti-CD69; Rabbit, suitable for IHC, reacts with human, mouse, rat
7) Anti-NKG2D; Rabbit, suitable for IHC, reacts with human, mouse, rat
8) Anti-Foxp3; Rabbit, suitable for IHC, reacts with human, mouse

Immunofluorescence Experiments:
1) Anti-CD31; rabbit; suitable for IHC-P; reacts with human, mouse, pig

Eukaryotic cell lines

Policy information about cell lines and Sex and Gender in Research

Cell line source(s)	Mouse skin melanoma cells B16-F10 (Cat. CRL-6475™, no record of the sex of the originating mouse) and mouse breast cancer cells 4T1 (Cat. CRL-2539™, female) were obtained from UArizona Cancer Center. Mouse skin melanoma cells B16-F10-Luc2 (Cat. CRL-6475-LUC2™, no record of the sex of the originating mouse), human breast adenocarcinoma cells Hela (Cat. CRM-CCL-2™, female) , human pancreatic adenocarcinoma cells Panc02 (Cat. CRL-2553™, female) and mouse macrophage cells RAW 264.7 (Cat. TIB-71™, male) were obtained from American Type Culture Collection (ATCC).
Authentication	Cell lines were used without any modification once received from respective suppliers and therefore were not authenticated.
Mycoplasma contamination	All cell lines were regularly tested for mycoplasma contamination and no mycoplasma contamination was observed.
Commonly misidentified lines (See ICLAC register)	None of the cell lines used are listed in the ICLAC list.

Animals and other research organisms

Policy information about studies involving animals; ARRIVE guidelines recommended for reporting animal research, and Sex and Gender in Research

Laboratory animals	5 weeks old, female C57BL/6 mice were purchased from Jackson laboratory. Mice were housed in Standard Individually Ventilated Caging (IVC). The Light cycle is 12/12 – 12 hours light/12 hours dark with 7am on-7pm off. The Temperature is maintained between 68°-72°F and the humidity is between 30-70% per the NIH Guide.
Wild animals	The study did not involve wild animals.
Reporting on sex	To ensure gender uniformity, the mice used in this study were female due to risk of more fighting in male colonies. Still, here analysis

is compared with or without treatment, so the sex of the host is considered less important.

Field-collected samples: No Fileld-collected sample were used in this study

Ethics oversight: All animal experiments were approved by The University of Arizona Institutional Animal Care and Use Committee (IACUC)

Note that full information on the approval of the study protocol must also be provided in the manuscript.

Plants

Seed stocks: N/A

Novel plant genotypes: N/A

Authentication: N/A

Flow Cytometry

Plots

Confirm that:

- [x] The axis labels state the marker and fluorochrome used (e.g. CD4-FITC).
- [ ] The axis scales are clearly visible. Include numbers along axes only for bottom left plot of group (a 'group' is an analysis of identical markers).
- [ ] All plots are contour plots with outliers or pseudocolor plots.
- [ ] A numerical value for number of cells or percentage (with statistics) is provided.

Methodology

Sample preparation: For tissue sample, the tissue was first mechanically disrupted from mice and divided into small pieces and homogenized in cold DMEM medium to form single cell suspensions in the presence of collagenase type I. Samples were further filtered through a 70 µm cell strainer with red blood cells lysed by ACK lysis buffer. Detailed methods for sample preparation were provided under "Methods: Flow cytometry analysis"

Instrument: BD FACSCanto II (BD FACSDiva Software, version 8.01)

Software: BD FACSDiva Software (version 8.01) was used for data collection, FlowJo software (version 10.0.7, TreeStar, USA, 2014) was used for data analysis.

Cell population abundance: No sorting was performed.

Gating strategy: Generally, cells was first gated on FSC/SSC. Singlet cells were usually gated using FSC-H and FSC-A. Live cells were gated using Zombie. Detailed gating strategy were provided under Supplementary Figures 20, 21 and 22.

- [x] Tick this box to confirm that a figure exemplifying the gating strategy is provided in the Supplementary Information.
